# An isolable catenane consisting of two Möbius conjugated nanohoops

**DOI:** 10.1038/s41467-018-05498-6

**Published:** 2018-08-02

**Authors:** Yang-Yang Fan, Dandan Chen, Ze-Ao Huang, Jun Zhu, Chen-Ho Tung, Li-Zhu Wu, Huan Cong

**Affiliations:** 1 Key Laboratory of Photochemical Conversion and Optoelectronic Materials, Technical Institute of Physics and Chemistry; School of Future Technology, University of Chinese Academy of Sciences, Chinese Academy of Sciences, Beijing, 100190 China; 2 State Key Laboratory of Physical Chemistry of Solid Surfaces and Collaborative Innovation Center of Chemistry for Energy Materials (iChEM), Fujian Provincial Key Laboratory of Theoretical and Computational Chemistry, and Department of Chemistry, College of Chemistry and Chemical Engineering, Xiamen University, Xiamen, 361005 China

## Abstract

Besides its mathematical importance, the Möbius topology (twisted, single-sided strip) is intriguing at the molecular level, as it features structural elegance and distinct properties; however, it carries synthetic challenges. Although some Möbius-type molecules have been isolated by synthetic chemists accompanied by extensive computational studies, the design, preparation, and characterization of stable Möbius-conjugated molecules remain a nontrivial task to date, let alone that of molecular Möbius strips assembling into more complex topologies. Here we report the efficient synthesis, crystal structure, and theoretical study of a catenane consisting of two fully conjugated nanohoops exhibiting Möbius topology in the solid state. This work highlights that oligoparaphenylene-derived nanohoops, a family of highly warped and synthetically challenging conjugated macrocycles, can not only serve as building blocks for interlocked supermolecular structures, but also represent a new class of compounds with isolable Möbius conformations stabilized by non-covalent interactions.

## Abstract (web summary)

Molecules exhibiting Möbius topology are fascinating but challenging synthetic targets. Here, the authors report the elegant synthesis and crystal structure of a catenane formed from two fully conjugated, interlocked Möbius nanohoops, and use theoretical calculations to understand its conformational stability and aromaticity.

## Introduction

Molecular architectures with fascinating structures have attracted considerable interest across the scientific community for decades. The never-ending pursuits for exploring the frontiers of chemical synthesis have turned many such molecules, from once imaginary pictures or theoretical models, into tangible substances^1,2^. In particular, conjugated molecules displaying Möbius topology are intriguing targets because of their stunning structures and unique aromaticity^3–6^.

**Fig. 1:**
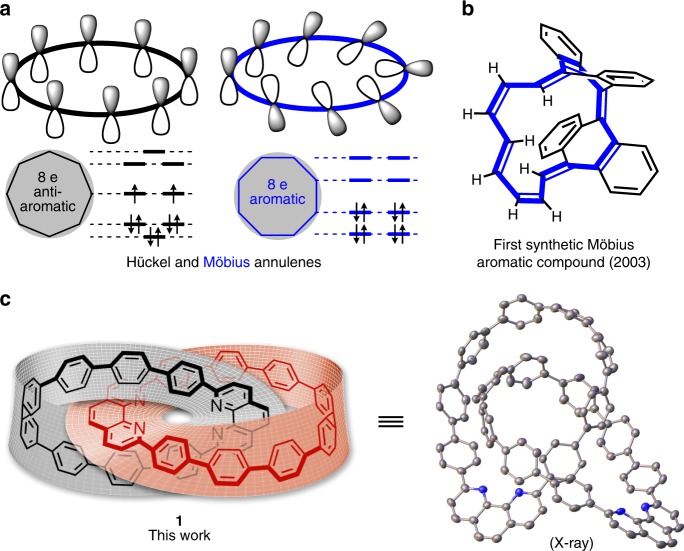
Molecules exhibiting Möbius topology. **a** Comparison of theoretical models for Hückel and Möbius aromaticity (applied to the example of cyclooctatetraene). **b** Herges and co-workers prepared a polyene-derived compound that has been regarded as the first isolated Möbius aromatic macrocycle. **c** Catenane **1** consisting of Möbius-conjugated nanohoops, with its X-ray crystal structure shown with 30%-probability ellipsoids. Hydrogen atoms and solvent molecules are omitted for clarity

In complement to the conventional Hückel’s rule for determining aromaticity, Heilbronner^7^ proposed in 1964 that cyclic-conjugated molecules displaying Möbius conformation are aromatic when their cyclic *π* systems contain 4*n* delocalized electrons (*n* is a positive integer, Fig. 1a). This pioneering prediction was validated in 2003 with the first synthetic Möbius aromatic compound (Fig. 1b)^8^. Recently, significant progress in synthesizing Möbius-type molecules has been achieved with the isolation of a number of expanded porphyrin derivatives^9^. The aforementioned compounds showcase creative structural designs relying on well-balanced tuning of molecular strain and rotational flexibility to secure Möbius conformations.

Our target catenane **1** (Fig. 1c) features oligoparaphenylene-derived nanohoops as building blocks^10^. Marked by considerable synthetic complications, these highly distorted nanohoops^11^ can adopt intriguing conformations promoted by stereochemical “gearing effect”^12,13^ and non-covalent *π*-*π* interaction^14–16^. We speculate that such promotional factors would be magnified within the [2] catenane structure to stabilize rotational conformers due to the proximity of the interlocked macrocycle components^17,18^. In addition, incorporating a phenanthroline moiety in each monomeric nanohoop would reinforce molecular rigidity, and facilitate catenane assembling using Sauvage’s copper(I)-templated method^19,20^.

## Results

### Synthesis and characterization

**Fig. 2:**
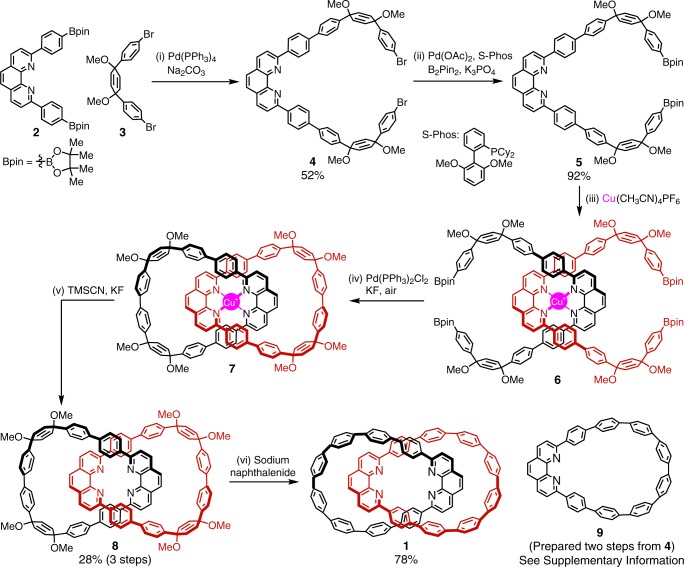
Synthetic routes to catenane **1**. The reaction conditions were as follows: (i) **2** (1 equiv), **3** (2.5 equiv), Pd(PPh_3_)_4_ (10 mol%), Na_2_CO_3_ (20 equiv), and toluene/water, 100 ^o^C, 24 h. (ii) **4** (1 equiv), B_2_pin_2_ (4 equiv), Pd(OAc)_2_ (10 mol%), S-Phos (20 mol%), K_3_PO_4_ (6 equiv), and 1,4-dioxane, 85 ^o^C, 24 h. (iii) **5** (1 equiv), Cu(CH_3_CN)_4_PF_6_ (0.5 equiv), and THF, 25 ^o^C, 10 min. (iv) Crude **6** in solution, Pd(PPh_3_)_2_Cl_2_ (1 equiv), KF (20 equiv), and THF/H_2_O, 25 ^o^C, 24 h. (v) Crude product **7**, TMSCN (10 equiv), KF (15 equiv), and CH_2_Cl_2_/CH_3_CN/H_2_O, 25 ^o^C, 6 h. (vi) **8** (1 equiv), THF, and freshly prepared sodium naphthalenide (0.2 M solution in THF, 15 equiv), –78 ^o^C, 1.5 h. Bpin boronic acid pinacol ester, Me methyl, Ph phenyl, Cy cyclohexyl, TMS trimethylsilyl, OAc acetate, THF tetrahydrofuran

The synthesis of **1** commenced with phenanthroline-derived arylboronic ester **2** and aryl bromide **3** (Fig. 2), both of which could be readily prepared from simple starting materials. Suzuki-Miyaura cross-coupling between **2** and excess **3** proceeded smoothly to afford product **4**, which was further converted to an extended bis(arylboronic ester) **5** through Miyaura borylation. With the precursor for catenane synthesis in hand, we next mixed **5** and a copper hexafluorophosphate salt with a 2:1 molar ratio. Upon the formation of copper(I) complex **6**, addition of a palladium catalyst and a fluoride base under the exposure of air initiated Jasti’s oxidative homocoupling macrocyclization^21^, generating copper-containing interlocked molecule **7**. Without further purification, crude compound **7** was subjected to the demetalation step in the presence of in situ released cyanide. The resulting metal-free catenane **8** was carefully reduced using sodium naphthalenide under cryogenic conditions^22^, producing the target catenane **1**. The interlocked structures of **1** and its precursor **8** were characterized by nuclear magnetic resonance (NMR) and mass spectrometry analyses (Supplementary Fig. 6, 9–11). The observed symmetrical ^1^H NMR spectra indicate fast conformational changes within the catenane structures in solution. Additionally, **1** exhibits much better solubility in common organic solvents compared to the monomeric nanohoop **9**, likely due to the reduced intermolecular *π*-*π* stacking in the solid state.

We obtained single crystals by slow diffusion of hexanes into a chloroform solution of **1**, and unambiguously confirmed its solid-state structure by X-ray crystallography (Fig. 1c). In addition to the validation of the mechanically linked feature, it was intriguing to observe that both conjugated nanohoops within **1** exclusively adopt Möbius topology in the solid state. Each monomeric nanohoop deviates from C_2_ symmetry, likely resulting from non-covalent interaction between the monomers. The average distance between a nitrogen atom and the furthest *ipso*-carbon within a monomeric nanohoop is 1.4 nm, and the width of one macrocycle monomer is measured as 1.1 nm. While the phenanthroline moieties remain almost flat, the oligoparaphenylene subunits are unevenly twisted, with torsional angles between neighboring aryl rings ranging from 2.6° to 55.6° (29.4° on average, Supplementary Fig. 12). With regard to the solid-state packing, the two monomeric nanohoops within a single molecule of **1** display a dihedral angle of 56°, and **1** adopts two opposite orientations that alternatively and parallelly organized in the crystal structure (Supplementary Fig. 13).

### Computational studies

**Fig. 3:**
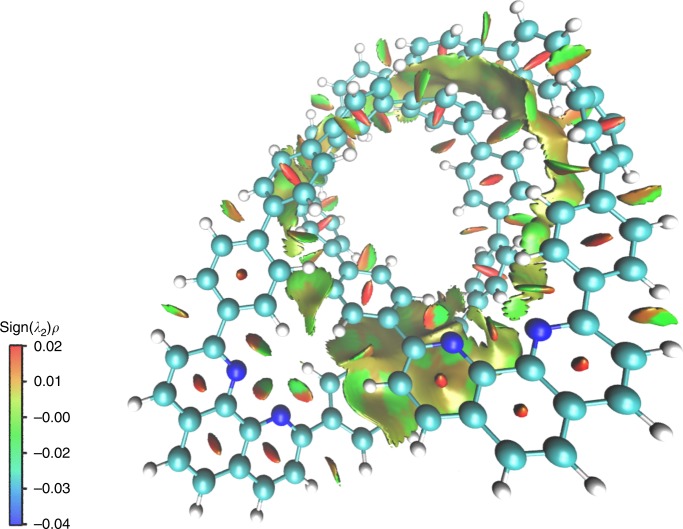
Gradient isosurface colored according to values of sign(*λ*_2_)*ρ* of the energy minimized structure of catenane **1**. An isovalue of 0.7 a.u. is applied. The color scale ranges from −0.04 (blue) to 0.02 (red) a.u. Large, negative values of sign(*λ*_2_)*ρ* indicate attractive interaction. Large, positive values reflect strong non-bonded overlapping which is generally resulted from steric repulsion. Values near zero correspond to the magnitude of van der Waals interaction

We performed computational studies on the structures optimized from the crystal structure of **1**. To probe the magnitude of intramolecular interaction within the catenane structure, the sign(*λ*_2_)*ρ* function was employed to examine the real-space non-covalent interaction (NCI) between the two monomeric nanohoops (Fig. 3)^23^. The low-gradient, low-density isosurfaces located at the centers of benzene rings are signs of typical non-bonded interaction commonly observed in small rings. The bent sheets exhibit a mix of green and orange areas, indicating the coexistence of van der Waals interaction and steric repulsion between the monomers. Such an overlap in bent sheets resembles to the isosurface in the benzene dimer^23^ as well as biphenyl dimer (Supplementary Fig. 14), where *π*–*π* stacking leads to a portion of strong non-bonded interaction. On the basis of energy decomposition analysis (EDA), the overall interaction energy between the monomers results in –84.0 kcal mol^−1^ in favor of stabilizing the catenane structure (Supplementary Table 3)^24^.

**Fig. 4:**
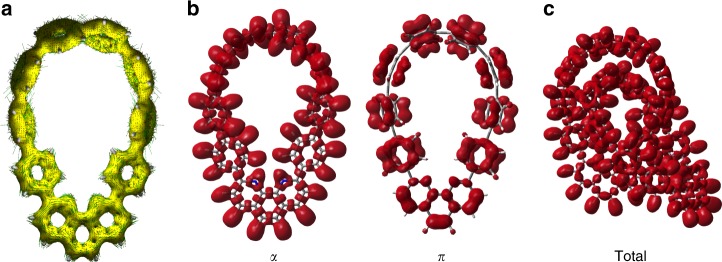
Electron delocalization evaluation of the energy minimized structures of catenane **1** and monomer **9**. **a** The ACID plot for monomer **9**. **b** ELF domains dissected into *σ* and *π* contributions in monomer **9**. **c** the ELF domains in catenane **1**. The vector of external magnetic field used to induce currents is parallel to the line of sight. Small green arrows are computed current density vectors. The *π* molecular orbitals are listed in Supplementary Table 4. Isovalues for ACID and ELF surfaces are 0.050 a.u. and 0.65 a.u., respectively

As the anisotropy of the induced current density (ACID) can simulate the density and direction of the induced ring current in a molecular system under an external magnetic field^25,26^, we computed the ACID plot for monomer **9** to examine its aromaticity (Fig. 4a). Clockwise currents can be observed in each individual benzene ring as well as the phenanthroline subunit, indicating local aromaticity in these moieties. Along the bridging C–C bonds, the current densities are comparable to those in the benzene rings. Specifically, we examined the critical isosurface value (CIV), an indicator of conjugation/delocalization^25^. The results show that the degree of conjugation between adjacent rings is comparable to that in benzene moieties (Supplementary Fig. 15).

To further investigate the electron delocalization over the monomer nanohoop **9** and catenane **1**, we carried out electron localization function (ELF) analysis which is also a robust tool for the evaluation of aromaticity^27,28^. The *σ*- and *π*-electron contributions to the ELF are separated for the monomer **9** (Fig. 4b). The ELF_*π*_ domains display the delocalization of *π* electrons in all benzene and phenanthroline moieties. Note that the ELF_*π*_ bifurcation values for the bridging C–C bonds are less than 0.3. According to a previous study on a series of polybenzenoid hydrocarbons, an ELF_*π*_ bifurcation value smaller than 0.65 should not be attributed to C–C delocalized *π* bonds^27^. However, the ELF_*σ*_ bifurcation values range from 0.573 to 0.679 for the bridging C–C bonds (Supplementary Fig. 16), which are close to the value (0.692) in benzene computed at the same level of theory. Therefore, the delocalization of the bridging C–C bonds is mainly attributed to the *σ* electrons, similar to the previous arguement by Shaik, Hiberty, and co-workers that the *σ*-framework of benzene leads to the observed *D*_6*h*_ symmetric structure^29^. The overall ELF_total_ domains in catenane **1** (Fig. 4c) suggest that the electron delocalization along the bridging C–C bonds are comparable to that over the aromatic moieties (Supplementary Fig. 17). Thus the monomer **9** and catenane **1** could be considered aromatic because each individual ring is aromatic and the bridging C–C bonds are significantly delocalized.

## Discussion

In summary, we report an efficient, precise synthesis of catenane **1** consisting of two mechanically linked, fully conjugated nanohoops. Its crystal structure exhibits unusual topology, representing a rare case of stabilized Möbius twisted *π*-system in the solid state. Theoretical calculations reveal the aromaticity in the monomer nanohoop as well as a strong driving force for the stabilization of catenane **1** through non-covalent *π*-*π* interaction. This work illustrates a productive combination of rational design, synthetic execution, crystallographic analysis, and computational investigation, which would ultimately inspire us and others along the march of discovering challenging and complex molecular architectures.

### Data availability

All the data supporting the findings of this study are available within the article and its Supplementary Information files or from the authors on reasonable request.

Supplementary crystallographic information files, which include structure factors, have been deposited with the Cambridge Crystallographic Data Centre (CCDC) as deposition number CCDC: 1835146, for compound **1**. The data files can be obtained free of charge from http://www.ccdc.cam.ac.uk/data_request/cif.

## Electronic supplementary material

Supplementary Information
