# Supplementary material for: An isolable catenane consisting of two Möbius conjugated nanohoops
Source: Nat Commun. 2018 Aug 2;9:3037. doi: 10.1038/s41467-018-05498-6 (PMC6072741; doi:10.1038/s41467-018-05498-6)
Supplement: Supplementary file 1 — Supplementary Information [file 41467_2018_5498_MOESM1_ESM.pdf]

Supplementary Information

An Isolable Catenane Consisting of Two Möbius Conjugated Nanohoops

Fan et al.

Supplementary Methods

General Information

^{1}H and ^{13}C NMR spectra were recorded on Bruker 400 MHz instruments, and are internally referenced to residual protio solvent signals (for $CDCl_3$, referenced at 7.26 and 77.16 ppm, respectively, see: Gottlieb, H. E.; Kotlyar, V.; Nudelman, A. *J. Org. Chem.* **1997**, *62*, 7512). Data for ^{1}H NMR are reported as follows: chemical shift (δ ppm), integration, multiplicity (br = broad, ovrlp = overlapping, s = singlet, d = doublet, t = triplet, q = quartet, m = multiplet), and coupling constant (Hz) when applicable. All ^{13}C NMR spectra were recorded with complete proton decoupling. Infrared spectra were recorded on a Varian 3100 FT-IR. High-resolution mass spectra were obtained on an AB Sciex MALDI-TOF/TOF5800 mass spectrometer using TCNQ matrix.

All reactions were carried out using flame-dried glassware under a nitrogen atmosphere unless otherwise noted. Analytical thin layer chromatography (TLC) was performed using 0.25 mm silica gel 60-F plates. Flash chromatography was performed using 200-400 mesh silica gel. HPLC-grade tetrahydrofuran, methylene chloride, diethyl ether, toluene, and hexane were purified and dried by passing through a PURE SOLV® solvent purification system (Innovative Technology, Inc.). Commercially available anhydrous solvents were used as received without further purification. Chemical reagents were purchased from Strem, Acros, Energy Chemicals, J&K, and Alfa Aesar, and were used as received.

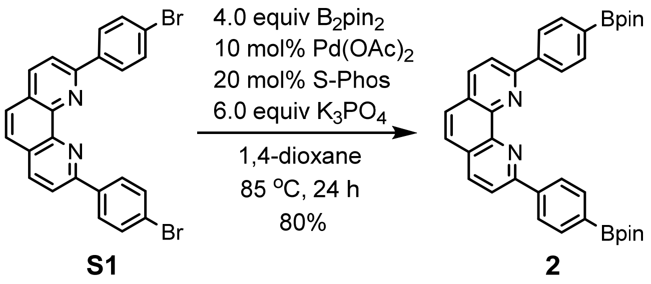

Supplementary Figure 1. Synthesis of compound **2**.

In a nitrogen-filled glove box, compound **S1** (Prepared according the literature: Sauvage, J.-P. *et al J. Am. Chem. Soc.* **2003**, *125*, 5717, 1.47 g, 3.00 mmol, 1.00 equiv), B_2pin_2 (3.05 g, 12.0 mmol, 4.00 equiv), $Pd(OAc)_2$ (66.0 mg, 0.300 mmol, 10 mol%), S-Phos (246 mg, 0.600 mmol, 20 mol%), anhydrous K_3PO_4 (3.82 g, 18.0 mmol, 6.00 equiv), and anhydrous 1,4-dioxane (26 mL) were sequentially added to a 75 mL glass heavy-wall pressure vessel (Beijing Synthware P170002) equipped with a stir bar. The vessel was capped, transferred out of glove box, and stirred at 85 °C for 24 h. The mixture was cooled to room temperature and filtered through a short pad of silica gel with the aid of 10:1 CH_2Cl_2/Et_3N (220 mL). The solvents were removed under reduced pressure. The resulting solid was recrystalized using hot hexane to afford compound **2** (1.39 g, 80% yield) as a yellow solid.

Compound 2

^{1}H NMR (400 MHz, $CDCl_3$) δ 8.50 (d, *J* = 8.2 Hz, 4H), 8.31 (d, *J* = 8.4 Hz, 2H), 8.19 (d, *J* = 8.4 Hz, 2H), 8.03 (d, *J* = 8.2 Hz, 4H), 7.79 (s, 2H), 1.40 (s, 24H);

^{13}C NMR (100 MHz, $CDCl_3$) δ 156.7, 146.3, 141.9, 137.0, 135.4, 128.2, 126.9, 126.3, 120.2, 84.0, 25.1;

IR (film): 2979, 1609, 1412, 1519, 1361, 1145, 1091, 846, 657 cm^{-1};

HRMS (MALDI): $[M+H]^+$ calcd for $C_{36}H_{39}B_2N_2O_4$ 585.3090, found 585.3101.

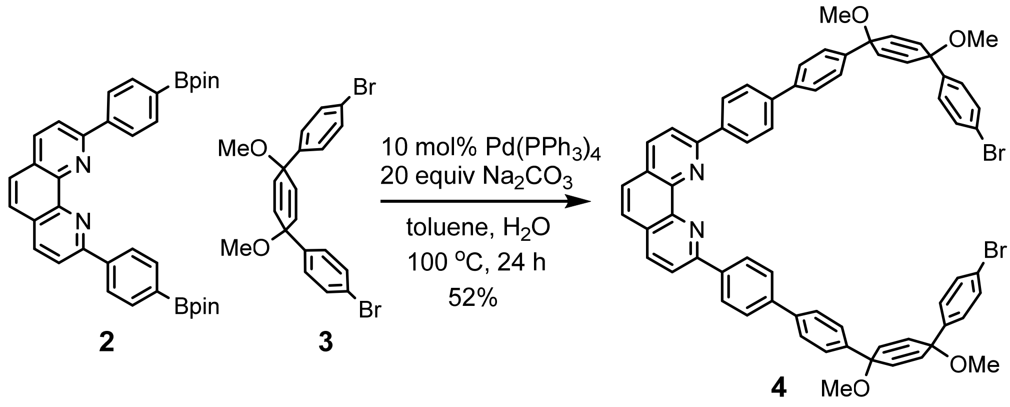

Supplementary Figure 2. Synthesis of compound **4**.

In a nitrogen-filled glove box, compound **2** (583 mg, 1.00 mmol, 1.00 equiv), compound **3** (Prepared according the literature: Jasti, R. *et al J. Org. Chem.* **2012**, *77*, 6624, 1125 mg, 2.50 mmol, 2.50 equiv), $Pd(PPh_3)_4$ (116 mg, 0.100 mmol, 10 mol%), anhydrous Na_2CO_3 (2120 mg, 20.0 mmol, 20.0 equiv), and anhydrous toluene (25 mL) were added to a 100 mL round-bottom flask equipped with a stirbar. The flask was capped with a rubber septum, and sferred out of glove box. Degassed water (8.00 mL) was then added to the flask via a syringe and the flask was connected to a nitrogen balloon via a needle. The reaction mixture was stirred at 100 °C for 24 h. The mixture was cooled to room temperature and filtered through a short pad of silica gel with the aid of 10:1 CH_2Cl_2/Et_3N (330 mL). The solvents were removed under reduced pressure. Purification using silica gel column chromatography (petroleum ether/$CHCl_3/Et_3N$ = 70:25:5) afforded compound **4** (557 mg, 52% yield) as a white solid.

Compound 4

^{1}H NMR (400 MHz, $CDCl_3$) δ 8.57 (d, *J* = 8.3 Hz, 4H), 8.31 (d, *J* = 8.4 Hz, 2H), 8.19 (d, *J* = 8.4 Hz, 2H), 7.85 (d, *J* = 8.3 Hz, 4H), 7.79 (s, 2H), 7.71 (d, *J* = 8.3 Hz, 4H), 7.50 (d, *J* = 8.3 Hz, 4H), 7.47 (d, *J* = 8.5 Hz, 4H), 7.33 (d, *J* = 8.5 Hz, 4H), 6.20 (d, *J* = 10.2 Hz, 4H), 6.10 (d, *J* = 10.2 Hz, 4H), 3.48 (s, 6H), 3.45 (s, 6H);
^{13}C NMR (100 MHz, $CDCl_3$) δ 156.5, 146.3, 142.71, 142.66, 141.6, 140.1, 138.6, 137.0 133.9, 133.1, 131.6, 128.2, 128.1, 128.0, 127.6, 127.3, 126.6, 126.2, 121.8, 120.0, 74.8, 74.7, 52.2, 52.1;
IR (film): 3030, 2930, 1607, 1486, 1399, 1079, 1009, 823 cm^{-1};
HRMS (MALDI): $[M+H]^+$ calcd for $C_{64}H_{51}Br_2N_2O_4$ 1069.2210, found 1069.2192.

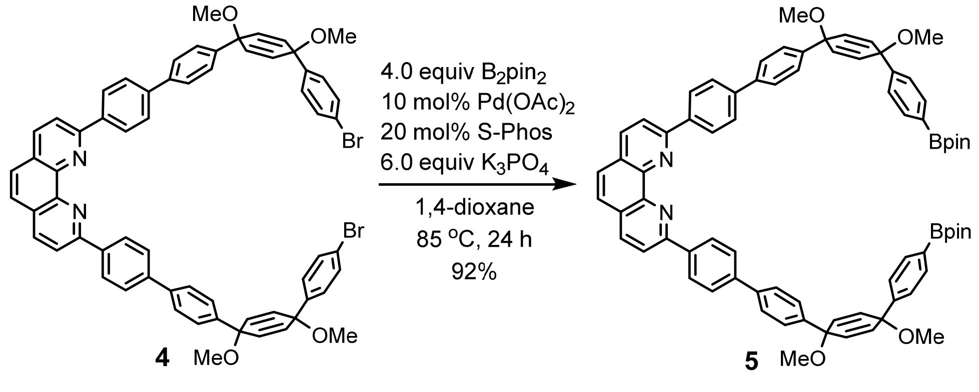

Supplementary Figure 3. Synthesis of compound **5**.

In a nitrogen-filled glove box, compound **4** (535 mg, 0.500 mmol, 1.00 equiv), B_2pin_2 (508 mg, 2.00 mmol, 4.00 equiv), $Pd(OAc)_2$ (11.2 mg, 0.0500 mmol, 10 mol%), S-Phos (41.1 mg, 0.100 mmol, 20 mol%),

anhydrous K_3PO_4 (254 mg, 1.20 mmol, 6.00 equiv), and anhydrous 1,4-dioxane (5 mL) were sequentially added to a 20 mL glass vial equipped with a stir bar. The vial was sealed with a teflon-lined septum cap, transferred out of glove box, and stirred at 85 °C for 24 h. The mixture was cooled to room temperature and filtered through a short pad of silica gel with the aid of 10:1 CH_2Cl_2/Et_3N (55 mL). The solvents were removed under reduced pressure. The resulting solid was recrystalized using hot hexane to afford compound **4** (536 mg, 92% yield) as a white solid.

Compound 5

^{1}H NMR (400 MHz, $CDCl_3$) δ 8.58 (d, J = 8.3 Hz, 4H), 8.32 (d, J = 8.4 Hz, 2H), 8.20 (d, J = 8.4 Hz, 2H), 7.86 (d, J = 8.3 Hz, 4H), 7.81 (d, J = 8.1 Hz, 4H), 7.79 (s, 2H), 7.70 (d, J = 8.3 Hz, 4H), 7.53 (d, J = 8.3 Hz, 4H), 7.47 (d, J = 8.1 Hz, 4H), 6.18 (d, J = 10.5 Hz, 4H), 6.14 (d, J = 10.5 Hz, 4H), 3.49 (s, 6H), 3.47 (s, 6H), 1.32 (s, 24H);

^{13}C NMR (100 MHz, $CDCl_3$) δ 156.5, 146.6, 146.4, 142.9, 141.7, 140.0, 138.5, 137.0, 135.1, 133.5, 128.2, 128.1, 127.6, 127.3, 126.6, 126.2, 125.5, 120.1, 83.9, 75.1, 74.9, 52.12, 52.11, 25.0;

IR (film): 3032, 2978, 1610, 1486, 1361, 1145, 1086, 828 cm^{-1};

HRMS (MALDI): $[M+H]^+$ calcd for $C_{76}H_{75}B_2N_2O_8$ 1165.5704, found 1165.5709.

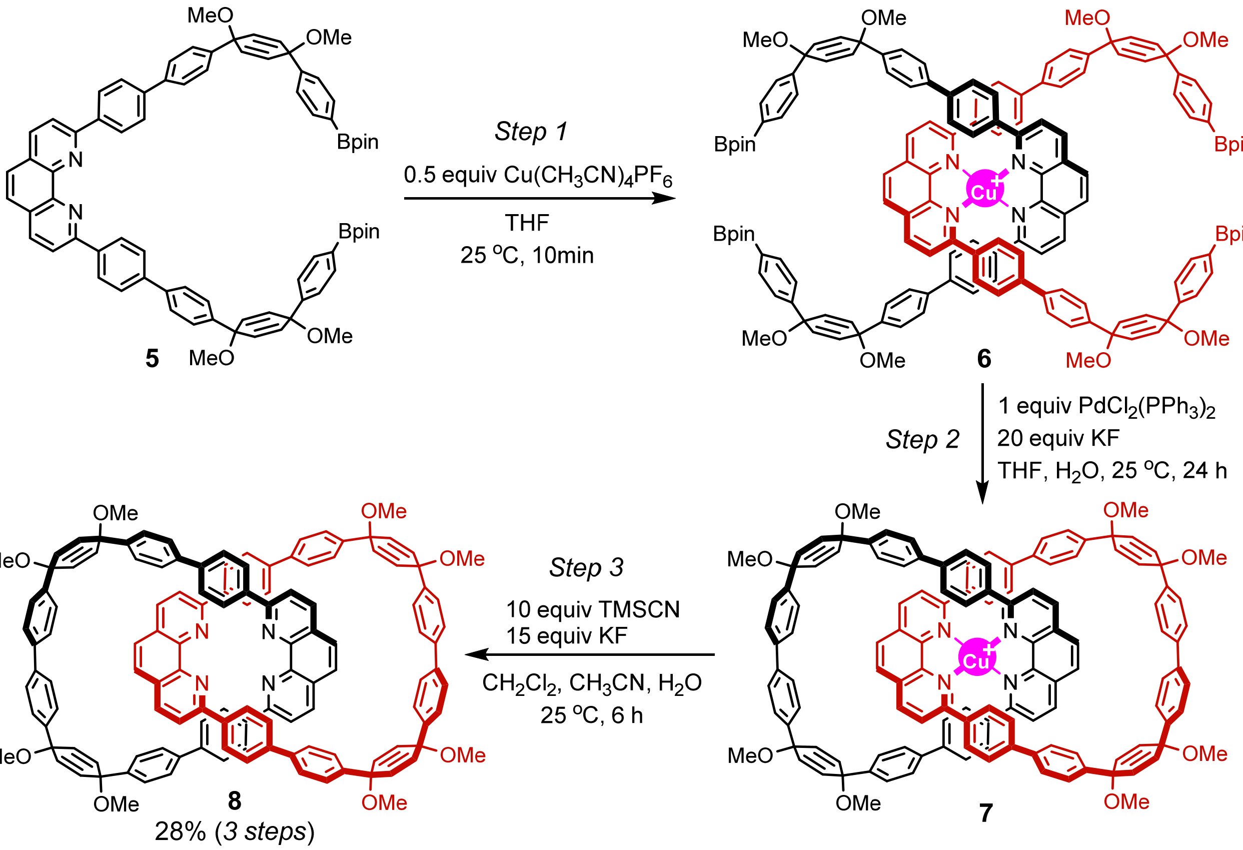

Supplementary Figure 4. Synthesis of compound **8**.

Step 1

In a nitrogen-filled glove box, compound **5** (117 mg, 0.100 mmol, 1.00 equiv), $Cu(CH_3CN)_4PF_6$ (18.6 mg, 0.050 mmol, 0.500 equiv), and anhydrous tetrahydrofuran (5 mL) were sequentially added to a 20 mL glass vial equipped with a stir bar. The vial was sealed with a teflon-lined septum cap, transferred out of glove box, and stirred at 25 °C for 10 min.

Step 2

The resulting dark red solution was transferred, with the aid of tetrahydrofuran (300 mL) in air, into a 500 mL round-bottom flask equipped with a stir bar. Then $Pd(PPh_3)_2Cl_2$ (70.0 mg, 0.100 mmol, 1.00 equiv), KF (116 mg, 2.00 mmol, 20.0 equiv), and H_2O (35 mL) were sequentially added to the flask. The flask was capped with a rubber septum and connected to an air balloon via a needle. The reaction mixture was stirred at 25 °C for 24 h. Then the solvents were removed under reduced pressure.

Step 3

The resulting crude mixture was transferred, with the aid of CH_2Cl_2 (10 mL) and CH_3CN (10 mL) in air, into a 100 mL round-bottom flask equipped with a stir bar. Then TMSCN (0.125ml, 1.00 mmol, 10.0 equiv), KF (58.0 mg, 1.50 mmol, 15.0 equiv), and H_2O (10 mL) was sequentially added to the flask. The flask was capped with a rubber septum and connected to an air balloon via a needle. The reaction mixture was stirred at 25 °C for 6 h. The reaction mixture was extracted with CH_2Cl_2 (20 mL × 3). The combined organic layers was washed with brine and dried over Na_2SO_4. (*Caution!* All cyanide-containing aqueous waste from this step should be carefully collected in a fume hood, and quenched by mixing with 5 mL 30% hydrogen peroxide prior to disposal.) The solvents were removed under reduced pressure. Purification using silica gel column chromatography (petroleum ether/$CHCl_3$/Et_3N = 65:30:5) afforded compound **8** (26.2 mg, 28% yield) as a white solid.

Compound 8

^{1}H NMR (400 MHz, $CDCl_3$) δ 8.54 (d, *J* = 8.4 Hz, 8H), 8.23 (d, *J* = 8.5 Hz, 4H), 8.13 (d, *J* = 8.5 Hz, 4H), 7.72 (s, 4H), 7.52 (d, *J* = 8.3 Hz, 8H), 7.45-7.49 (m, 16H), 7.43 (d, *J* = 8.3 Hz, 8H), 7.36 (d, *J* = 8.3 Hz, 8H), 6.12 (d, *J* = 10.3 Hz, 8H), 6.07 (d, *J* = 10.3 Hz, 8H), 3.40 (s, 12H), 3.35 (s, 12H);

^{13}C NMR (100 MHz, $CDCl_3$) δ 156.2, 146.3, 143.0, 142.9, 141.5, 140.03, 139.55, 138.5, 136.8, 133.6, 133.2, 128.02, 127.97, 127.54, 127.52, 127.3, 126.6, 126.4, 126.0, 119.8, 74.8, 74.4, 52.1, 52.0;

IR (film): 3031, 2927, 1605, 1487, 1398, 1175, 1082, 825 cm^{-1};

HRMS (MALDI): $[M+H]^+$ calcd for $C_{128}H_{101}N_4O_8$ 1821.7614, found 1821.7597.

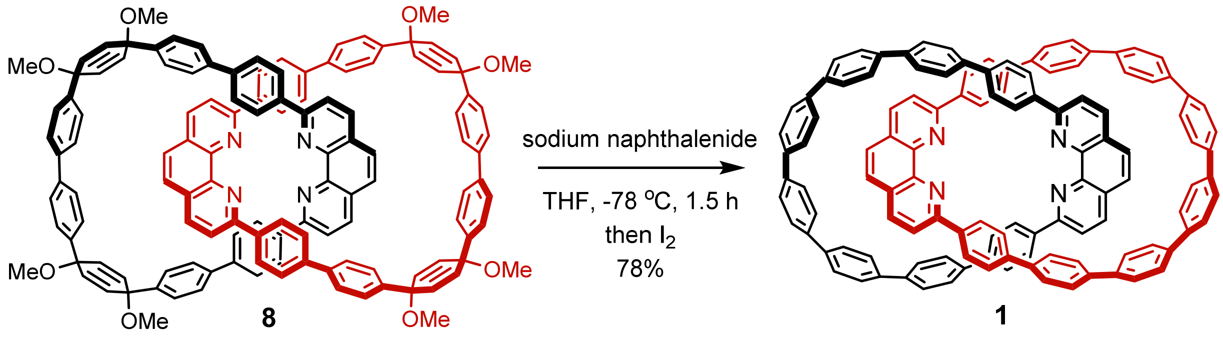

Supplementary Figure 5. Synthesis of compound **1**.

In a nitrogen-filled glove box, sodium metal (35.0 mg, 1.50 mmol), naphthalene (128 mg, 1.00 mmol), and anhydrous THF (5.00 mL) were added to a 20 mL glass vial equipped with a glass-coated stirbar (Note: using a teflon-coated stirbar would result in permanent black stain on the stirbar, although the reaction would not be affected). The vial was sealed with a teflon-lined septum cap, transferred out of glove box, and stirred at 25°C for 10 h, generating a dark green solution of sodium naphthalenide (0.2 M). Next, in the glove box, compound **8** (18.2 mg, 0.0100 mmol, 1.00 equiv) and anhydrous THF (2.1 mL) were added to a 40 mL glass vial equipped with a stirbar. The vial was sealed with a teflon-lined septum cap, transferred out of glove box, and cooled to -78 °C. The freshly prepared sodium naphthalenide solution (0.75 mL, 0.150 mmol, 15.0 equiv) was then added to the 40 mL vial dropwise via a syringe under nitrogen at -78 °C. The resulting mixture was

stirred for 1.5 h at -78 °C, before quenched at -78 °C with a 1 M solution of I_2 in THF (0.800 mL, 0.800 mmol) under nitrogen. The reaction mixture was warmed to room temperature, mixed with excess saturated aqueous sodium thiosulfate solution (approx. 10 mL), and extracted with CH_2Cl_2 (10 mL × 3). The combined organic layers were washed with brine and dried over Na_2SO_4. The solvents were removed under reduced pressure. Purification using silica gel column chromatography (petroleum ether/$CHCl_3$/Et_3N = 50:45:5) afforded compound **1** (12.9 mg, 78% yield) as a yellow solid.

Note: *The symmetrical ^1H NMR spectrum of compound* ***1*** *in solution indicates fast aryl-aryl single bond rotation and fast conformational changes between the two catenane components. Thus it should be noted that the stabilized conformation of* ***1*** *shows Möbius strip only when constrained in the solid state.*

Compound 1

^1H NMR (400 MHz, $CDCl_3$) δ 8.28 (d, J = 8.3 Hz, 4H), 8.09 (d, J = 8.1 Hz, 8H), 7.96 (d, J = 8.3 Hz, 4H), 7.82 (s, 4H), 7.36-7.28 (m, 32H), 7.24 (d, J = 8.3 Hz, 8H), 7.21 (d, J = 8.3 Hz, 8H), 7.11 (d, J = 8.1 Hz, 8H);

^{13}C NMR (100 MHz, $CDCl_3$) δ 157.6, 146.8, 142.3, 140.0, 139.2, 139.0, 138.3, 138.1, 137.8, 137.5, 136.8, 128.4, 128.1, 128.0, 127.7, 127.5, 127.4, 127.2, 126.2, 120.6;

IR (film): 2870, 2269, 1725, 1458, 1300, 1109, 951 cm^{-1};

HRMS (MALDI): $[M+H]^+$ calcd for $C_{120}H_{77}N_4$ 1573.6143, found 1573.6150.

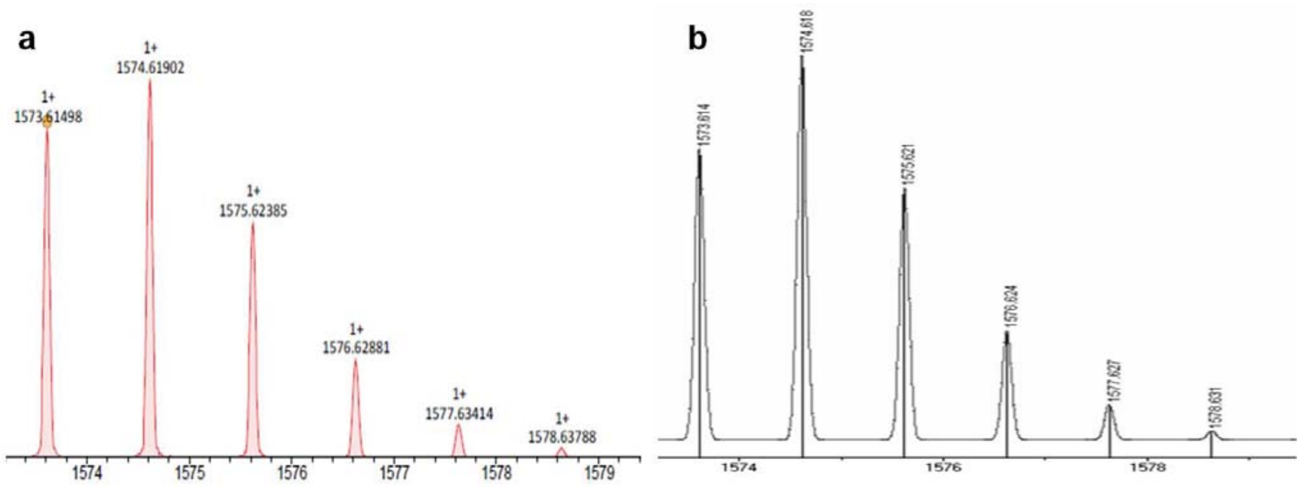

Supplementary Figure 6. a, Measured and **b**, simulated HRMS data for compound **1** ($[M+H]^+$ $C_{120}H_{77}N_4$).

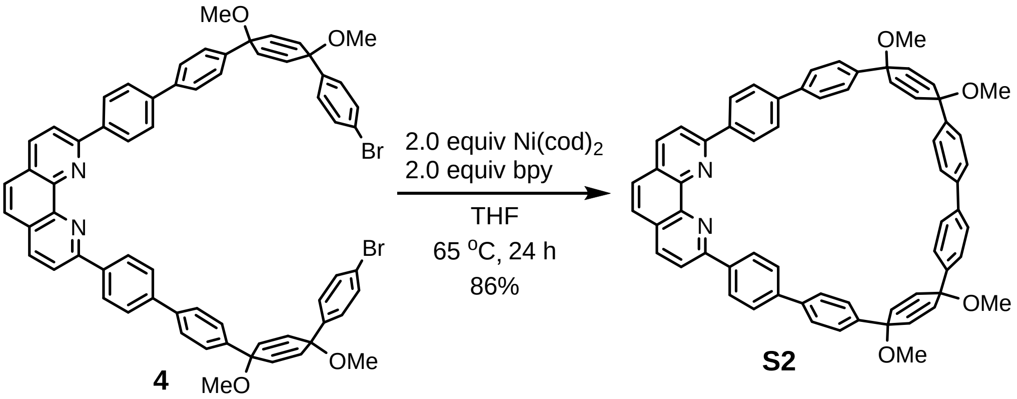

Supplementary Figure 7. Synthesis of compound S**2**.

In a nitrogen-filled glove box, $Ni(cod)_2$ (28.0 mg, 0.100 mmol, 2.00 equiv), 2,2'-bipyridyl (16.0 mg, 0.100 mmol, 2.00 equiv), and anhydrous THF (15 mL) were sequentially added to a 40 mL glass vial equipped with

a stir bar. The vial was sealed with a Teflon-lined septum cap, transferred out of glove box, and stirred at 50℃ for 0.5 h. The vial was then cooled to room temperature and moved back into the glove box. In the glove box, compound **4** (56.0 mg, 0.050 mmol, 1.00 equiv) and THF (30 mL) were added to a 150 mL glass heavy-wall pressure vessel (Beijing Synthware P170004) equipped with a stirbar. Next, the content in the 40 mL glass vial was transferred to the pressure vessel with aid of 5 mL THF via a syringe. The vessel was capped, transferred out of the glove box, and stirred at 65 ^{o}C for 24 h. The reaction mixture was cooled to room temperature and filtered through a short pad of silica gel with the aid of 10:1 $CHCl_3/Et_3N$ (100 mL). The solvent was removed under reduced pressure. The resulting solid was recrystalized using hot hexane to afford compound **S2** (39.0 mg, 86% yield) as a white solid.

Compound S2

^{1}H NMR (400 MHz, $CDCl_3$) δ 8.67 (d, *J* = 8.3 Hz, 4H), 8.33 (d, *J* = 8.4 Hz, 2H), 8.24 (d, *J* = 8.4 Hz, 2H), 7.91 (d, *J* = 8.3 Hz, 4H), 7.81 (s, 2H), 7.77 (d, *J* = 8.3Hz, 4H), 7.68 (d, *J* = 8.3 Hz, 4H), 7.59 (dd, *J* = 8.6 Hz, 8.5 Hz, 8H), 6.17 (s, 8H), 3.52 (s, 6H), 3.50(s, 6H);

^{13}C NMR (100 MHz, $CDCl_3$) δ 155.8, 146.2, 143.3, 143.0, 141.6, 140.0, 139.7, 138.2, 137.0, 133.7, 133.5, 128.1, 128.0, 127.5, 127.3, 127.2, 126.6, 126.1, 119.4, 75.1, 74.8, 52.3, 52.2;

IR (film): 3031, 2930, 2605, 2496, 1606, 1486, 1398, 1083, 824 cm^{-1};

HRMS (MALDI): $[M+H]^+$ calcd for $C_{64}H_{51}N_2O_4$ 911.3843, found 911.3860.

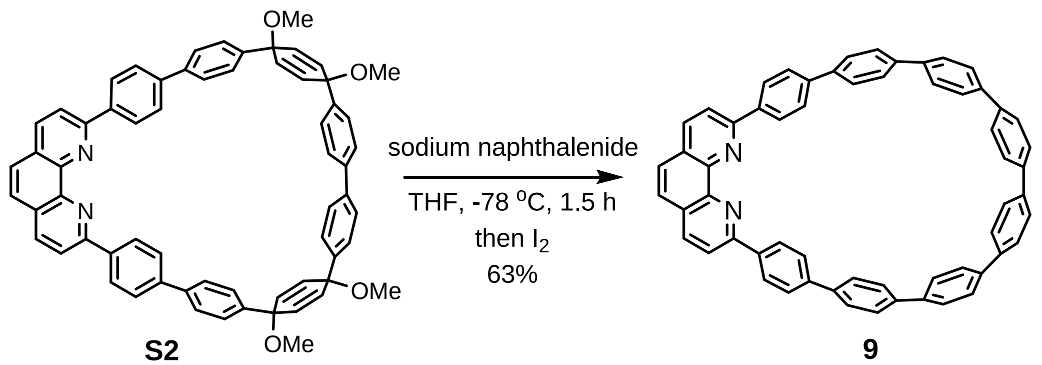

Supplementary Figure 8. Synthesis of compound **9**.

In a nitrogen-filled glove box, sodium metal (35.0 mg, 1.50 mmol), naphthalene (128 mg, 1.00 mmol), and anhydrous THF (5.00 mL) were added to a 20 mL glass vial equipped with a glass-coated stirbar (Note: using a teflon-coated stirbar would result in permanent black stain on the stirbar, although the reaction would not be affected). The vial was sealed with a teflon-lined septum cap, transferred out of glove box, and stirred at 25°C for 10 h, generating a dark green solution of sodium napthalenide (0.2 M).

Next, in the glove box, compound **S2** (27.3 mg, 0.030 mmol, 1.00 equiv) and anhydrous THF (6.0 mL) were added to a 40 mL glass vial equipped with a stirbar. The vial was sealed with a teflon-lined septum cap, transferred out of glove box, and cooled to -78 ^{o}C. The freshly prepared sodium napthalenide solution (1.50 mL, 0.300 mmol, 10.0 equiv) was then added to the 40 mL vial dropwise via a syringe under nitrogen at -78 ^{o}C. The resulting mixture was stirred for 1.5 h at -78 ^{o}C, before quenched at -78 ^{o}C with a 1 M solution of I_2 in THF (0.800 mL, 0.800 mmol) under nitrogen. The reaction mixture was warmed to room temperature, and mixed with excess saturated aqueous sodium thiosulfate solution (approx. 10 mL). The resulting suspension was separated by centrifugation (10000 rpm, 10 min). The yellow precipitate was washed (sonicated with 10 mL appropriate solvent for 3 min, followed by centrifugation at 10000 rpm for 10 min) with acetone twice and

then CH_2Cl_2 for three times, affording compound **9** (14.9 mg, 63% yield) as a yellow solid. Compound **9** is insoluble in most organic solvents and scarcely soluble only in CH_2Cl_2 and chloroform. Due to the poor solubility, ^{13}C NMR characterization of compound **9** was unsuccessful.

Compound 9

^{1}H NMR (400 MHz, $CDCl_3$) δ 8.38-8.32 (m, 6H), 8.14 (d, J = 8.0 Hz, 2H), 7.82 (s, 2H), 7.69-7.52 (m, 28H);
IR (film): 2920, 1636, 1399, 1306, 1261, 1129, 750, cm^{-1};
HRMS (MALDI): $[M+Na]^+$ calcd for $C_{60}H_{38}N_2Na$ 809.2927, found 809.2913.

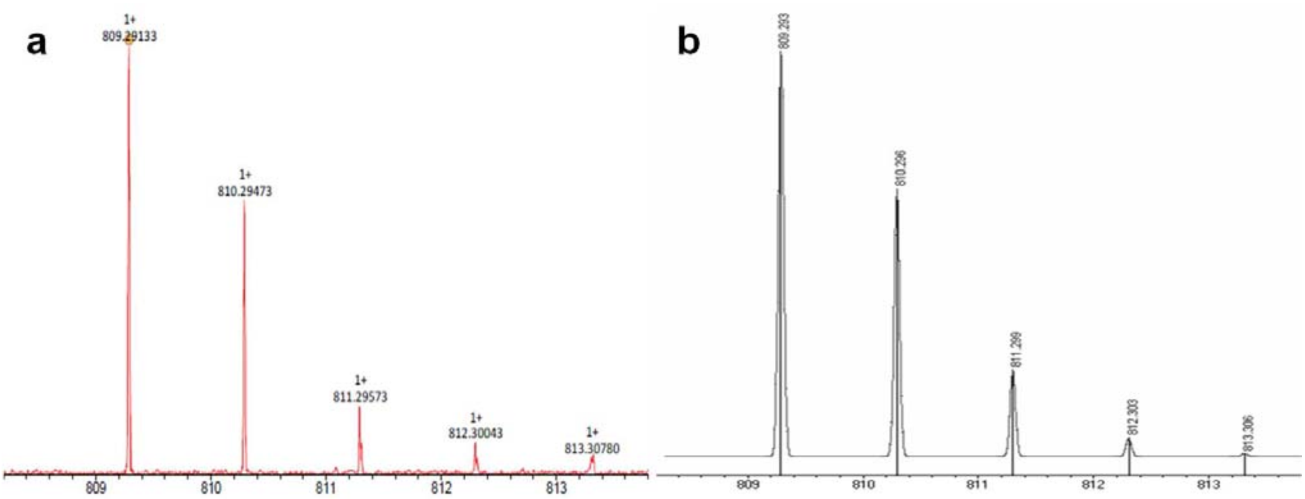

Supplementary Figure 9. a, Measured and **b**, simulated HRMS data for compound **9** ($[M+Na]^+$ $C_{60}H_{38}N_2Na$).

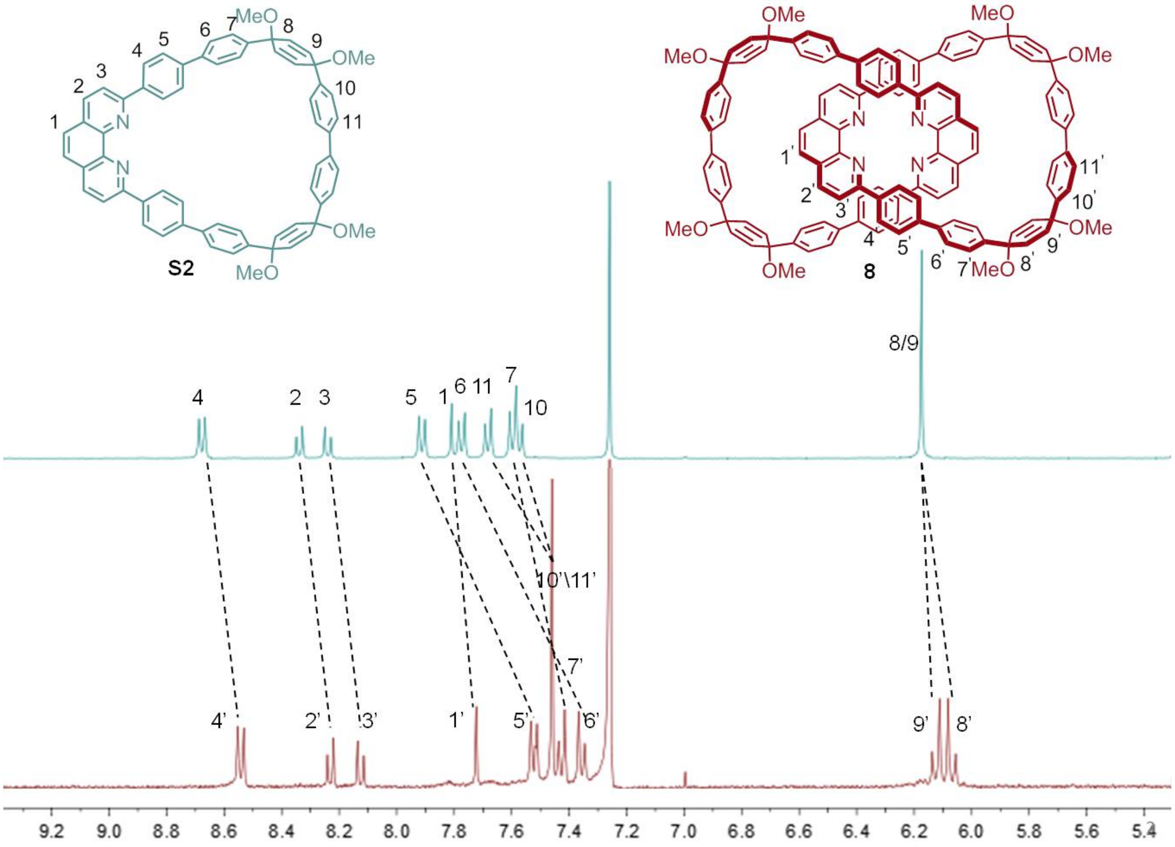

Supplementary Figure 10. Comparison of ^{1}H NMR data of compounds **S2** and **8**.

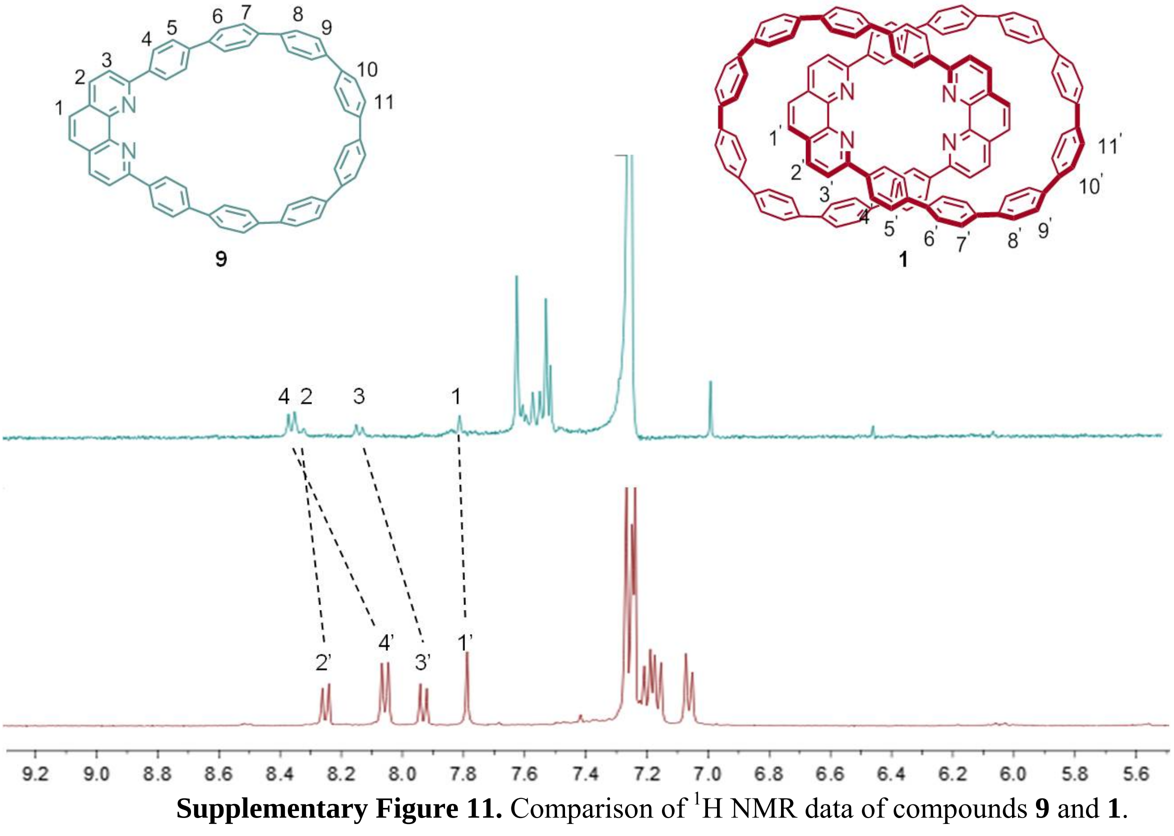

Supplementary Figure 11. Comparison of ^{1}H NMR data of compounds **9** and **1**.

Supplementary Discussion

X-ray Crystallography Analysis

X-ray crystallographic data were obtained on a MM007-HF CCD diffractometer with graphite-monochromated Mo Kα radiation (λ = 0.71073 Å, for compound **1**). Data collection was performed using the CrystalClear (Rigaku Inc., 2007) and reduction was performed using CrysAlisPro 1.171.39.9d (Rigaku OD, 2015). Multiscan absorption corrections were applied for all the datasets using the CrysAlisPro 1.171.39.9d (Rigaku Oxford Diffraction, 2015). The crystal structures were solved by direct methods (SHELXT-2014) and refined on F^2 by full-matrix least-squares techniques (SHELXL-2015) using all unique data. The unit of compound **1** contains three well-behaved $CHCl_3$ molecules, and a fourth one which could not be restrained properly, therefore, SQUEEZE algorithm was used.

Crystallographic data were deposited at the Cambridge Crystallographic Data Center (CCDC 1835146 for compound **1**). The data can be obtained free of charge from The Cambridge Crystallographic Data Centre via www.ccdc.cam.ac.uk/structures.

Compound 1

A single crystal suitable for X-ray crystallography was obtained by slow diffusion of hexanes into a chloroform solution of **1** at room temperature.

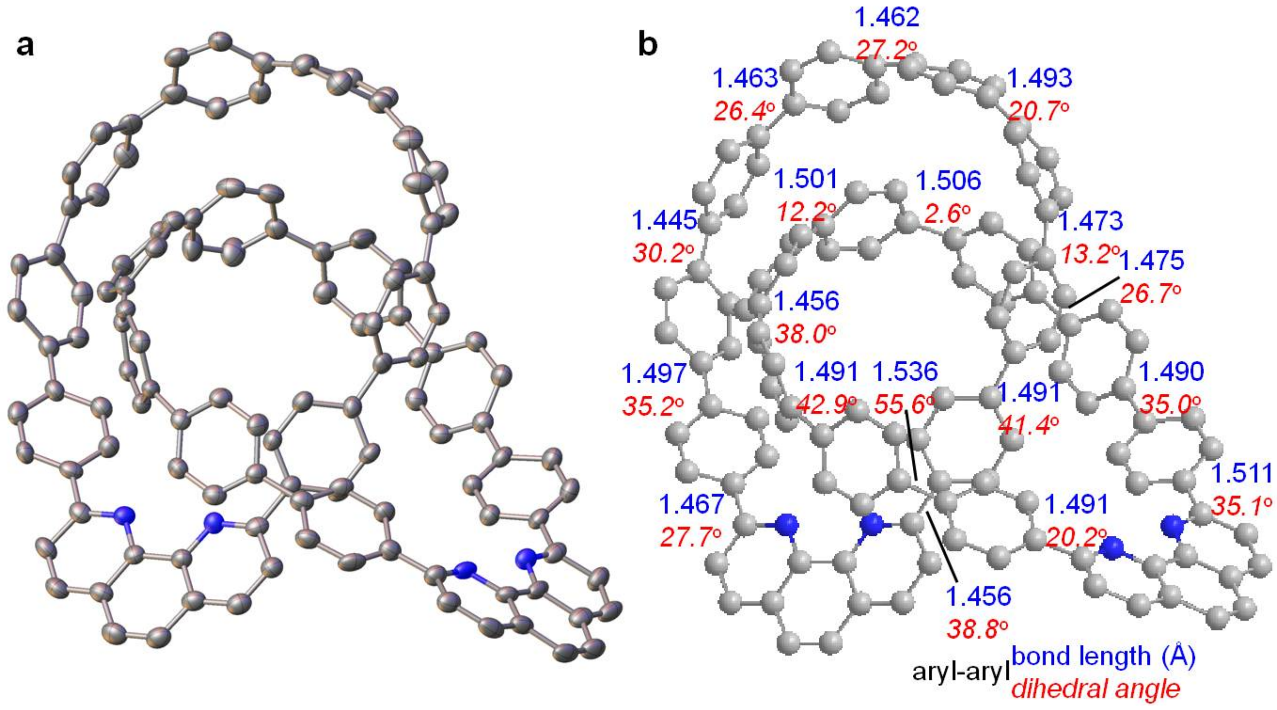

Supplementary Figure 12. a, ORTEP drawing of compound **1** with the thermal ellipsoids shown at a 30% probability. **b,** aryl-aryl bond lengths and dihedral angles within compound **1**. All solvents and protons were omitted for clarity.

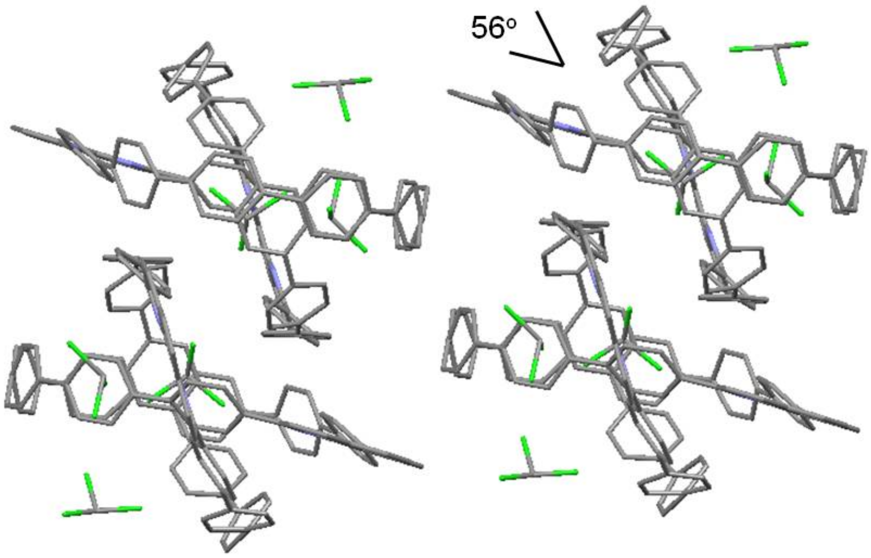

Supplementary Figure 13. Solid-state packing of compound **1**. All protons were omitted for clarity.

Supplementary Table 1. Crystal data and structure refinement for compound **1**.

Identification code	**1**	
Empirical formula	C_{123} H_{79} Cl_9 N_4	
Formula weight	1931.95	
Temperature	173.15 K	
Wavelength	0.71073 Å	
Crystal system	Triclinic	
Space group	P-1	
Unit cell dimensions	a = 15.0574(4) Å	α= 97.070(2)°.
	b = 17.0310(5) Å	β= 97.181(2)°.
	c = 20.5659(6) Å	γ = 102.380(2)°.
Volume	5049.4(3) $Å^3$	
Z	2	
Density (calculated)	1.271 Mg m^{-3}	
Absorption coefficient	0.303 mm^{-1}	
F(000)	1996	
Crystal size	0.154 x 0.115 x 0.064 mm^3	
Theta range for data collection	1.595 to 19.000°.	
Index ranges	-13<=h<=13, -15<=k<=15, -18<=l<=18	
Reflections collected	34023	
Independent reflections	8124 [R(int) = 0.0539]	
Completeness to theta = 25.200°	99.9%	
Absorption correction	Semi-empirical from equivalents	
Max. and min. transmission	1.00000 and 0.98155	
Refinement method	Full-matrix least-squares on F^2	
Data / restraints / parameters	8124 / 0 / 1225	
Goodness-of-fit on F2	1.488	

Final R indices [I>2sigma(I)]	$R_1 = 0.0897$, $wR_2 = 0.2310$
R indices (all data)	$R_1 = 0.1176$, $wR_2 = 0.2489$
Extinction coefficient	n/a
Largest diff. peak and hole	0.730 and -0.497 e.Å^{-3}

Supplementary Table 2. Atomic coordinates (x 10^4) and equivalent isotropic displacement parameters (Å^2 x 10^3) for compound **1**. U(eq) is defined as one third of the trace of the orthogonalized Uij tensor.

	x	y	z	U(eq)
N1	7823(5)	10052(4)	3822(6)	75(2)
N2	7399(5)	9705(4)	5053(4)	69(2)
C1	8035(7)	10216(5)	3223(6)	74(3)
C2	8937(7)	10586(5)	3149(5)	81(3)
C3	9603(7)	10860(5)	3712(8)	91(3)
C4	9385(7)	10729(5)	4352(7)	83(3)
C5	10054(6)	11050(5)	4966(8)	91(3)
C6	9797(8)	10906(6)	5549(7)	92(3)
C7	8916(7)	10460(6)	5598(7)	79(3)
C8	8673(7)	10280(6)	6207(7)	91(3)
C9	7808(7)	9811(6)	6245(5)	90(3)
C10	7180(7)	9524(6)	5638(6)	77(3)
C11	8246(7)	10149(5)	5008(6)	77(3)
C12	8476(7)	10309(5)	4374(6)	68(2)
C13	6228(7)	9045(6)	5630(5)	71(2)
C14	6011(7)	8483(7)	6055(5)	81(3)
C15	5095(7)	8048(5)	6027(4)	80(3)
C16	4374(6)	8197(6)	5581(5)	68(2)
C17	4617(7)	8755(5)	5158(4)	66(2)
C18	5520(7)	9164(5)	5177(4)	72(2)
C19	3407(6)	7717(6)	5526(4)	69(2)
C20	3181(7)	6876(6)	5577(4)	77(3)
C21	2283(7)	6420(5)	5387(4)	71(2)
C22	1568(6)	6777(6)	5149(4)	71(2)
C23	1782(7)	7607(7)	5148(4)	76(3)
C24	2696(7)	8079(5)	5332(4)	73(2)
C25	646(7)	6289(6)	4816(4)	76(3)
C26	568(7)	5510(7)	4476(6)	114(4)
C27	-184(8)	5135(6)	3993(6)	130(4)
C28	-886(6)	5517(7)	3839(6)	76(3)
C29	-878(7)	6206(7)	4237(5)	80(3)
C30	-137(7)	6600(6)	4716(5)	81(3)

	x	y	z	U(eq)
C31	-1424(7)	5282(7)	3152(6)	78(3)
C32	-1282(6)	4690(6)	2659(8)	89(3)
C33	-1431(8)	4716(6)	1997(7)	95(3)
C34	-1729(6)	5372(7)	1783(5)	73(3)
C35	-2055(7)	5870(7)	2226(7)	104(3)
C36	-1917(7)	5832(7)	2898(7)	104(3)
C37	-1462(6)	5671(6)	1166(5)	69(2)
C38	-1700(6)	6372(6)	958(5)	79(3)
C39	-1173(7)	6842(6)	591(5)	79(3)
C40	-391(7)	6647(6)	378(4)	70(3)
C41	-250(6)	5887(6)	499(5)	73(3)
C42	-765(7)	5417(6)	879(5)	80(3)
C43	373(6)	7229(6)	182(4)	61(2)
C44	665(6)	8024(6)	533(4)	65(2)
C45	1540(6)	8496(5)	522(4)	64(2)
C46	2159(6)	8187(5)	178(4)	63(2)
C47	1836(7)	7427(6)	-229(4)	70(2)
C48	953(7)	6969(5)	-228(4)	70(2)
C49	3157(6)	8568(6)	364(4)	63(2)
C50	3740(7)	8052(5)	521(4)	76(3)
C51	4617(6)	8353(6)	873(4)	75(3)
C52	4950(6)	9170(6)	1073(4)	62(2)
C53	4425(7)	9686(5)	848(4)	74(3)
C54	3549(7)	9386(6)	506(4)	68(2)
C55	5777(6)	9457(6)	1614(5)	72(3)
C56	5760(6)	9116(5)	2204(6)	74(3)
C57	6481(7)	9372(5)	2732(5)	71(2)
C58	7261(7)	9961(6)	2683(6)	73(3)
C59	7285(7)	10277(5)	2103(7)	81(3)
C60	6552(7)	10043(5)	1568(5)	78(3)
N1A	1215(6)	10490(4)	1399(3)	68(2)
N2A	2858(5)	10255(4)	2036(3)	65(2)
C1A	404(7)	10609(6)	1090(4)	72(3)
C2A	370(7)	11328(6)	831(4)	81(3)
C3A	1170(9)	11926(6)	894(5)	86(3)
C4A	1991(8)	11825(6)	1205(5)	72(2)
C5A	2811(8)	12446(6)	1290(5)	87(3)
C6A	3610(7)	12342(6)	1611(5)	84(3)
C7A	3659(8)	11605(6)	1870(5)	77(3)

	x	y	z	U(eq)
C8A	4471(7)	11481(6)	2201(5)	81(3)
C9A	4481(6)	10757(6)	2448(4)	77(3)
C10A	3642(7)	10151(6)	2346(4)	66(2)
C11A	2845(6)	10964(6)	1793(4)	66(2)
C12A	1987(7)	11081(6)	1452(4)	63(2)
C13A	3644(6)	9357(6)	2574(5)	69(2)
C14A	4158(6)	9290(6)	3168(5)	74(3)
C15A	4195(5)	8539(7)	3339(4)	71(3)
C16A	3697(6)	7826(6)	2933(5)	65(2)
C17A	3162(6)	7906(6)	2343(5)	69(2)
C18A	3126(6)	8648(7)	2169(4)	68(2)
C19A	3676(5)	6986(6)	3090(6)	66(2)
C20A	3643(5)	6341(7)	2592(4)	69(2)
C21A	3419(5)	5537(6)	2715(5)	70(2)
C22A	3211(6)	5338(6)	3307(6)	69(2)
C23A	3356(6)	5992(7)	3835(5)	78(3)
C24A	3573(6)	6788(6)	3717(5)	72(3)
C25A	2681(6)	4507(7)	3332(5)	75(3)
C26A	2512(7)	3858(7)	2832(5)	92(3)
C27A	1764(7)	3202(6)	2742(5)	90(3)
C28A	1104(8)	3173(6)	3164(6)	82(3)
C29A	1367(7)	3721(7)	3745(5)	91(3)
C30A	2154(8)	4356(6)	3830(5)	93(3)
C31A	114(7)	2730(5)	2935(6)	77(3)
C32A	-252(8)	2537(6)	2262(6)	87(3)
C33A	-1179(8)	2388(5)	2039(5)	82(3)
C34A	-1819(8)	2410(5)	2484(6)	77(3)
C35A	-1465(7)	2491(5)	3157(6)	78(3)
C36A	-530(8)	2662(5)	3373(5)	81(3)
C37A	-2711(7)	2555(6)	2258(6)	75(3)
C38A	-3065(7)	3092(7)	2661(5)	83(3)
C39A	-3681(7)	3521(6)	2420(6)	79(3)
C40A	-4001(6)	3420(7)	1739(7)	85(3)
C41A	-3785(7)	2772(7)	1348(5)	81(3)
C42A	-3162(7)	2340(6)	1591(6)	87(3)
C43A	-4349(6)	4090(6)	1459(6)	81(3)
C44A	-4704(6)	4648(8)	1871(5)	88(3)
C45A	-4699(6)	5405(7)	1707(6)	81(3)
C46A	-4365(6)	5668(7)	1151(6)	74(3)

	x	y	z	U(eq)
C47A	-4116(6)	5080(7)	711(5)	77(3)
C48A	-4100(6)	4313(7)	879(5)	80(3)
C49A	-4031(6)	6520(6)	1088(6)	75(3)
C50A	-3894(6)	6796(6)	484(5)	69(2)
C51A	-3307(7)	7532(7)	462(5)	74(3)
C52A	-2829(6)	8040(6)	1051(6)	68(2)
C53A	-3073(6)	7819(6)	1636(5)	74(3)
C54A	-3640(7)	7103(7)	1678(5)	76(3)
C55A	-2029(7)	8726(6)	1054(5)	68(2)
C56A	-1262(7)	8849(5)	1553(5)	74(3)
C57A	-482(6)	9449(6)	1547(5)	73(3)
C58A	-439(7)	9955(5)	1065(5)	69(2)
C59A	-1190(7)	9831(6)	567(5)	72(3)
C60A	-1968(7)	9221(7)	571(5)	79(3)
Cl1	2538(2)	2419(2)	5186(2)	117(1)
Cl2	2156(2)	2055(2)	6480(1)	113(1)
Cl3	4038(2)	2542(2)	6257(2)	145(1)
C61	2903(6)	1994(6)	5900(5)	101(3)
Cl4	1266(3)	7478(3)	3477(2)	181(2)
Cl5	1095(3)	6131(3)	2502(3)	206(2)
Cl6	351(4)	7412(3)	2184(3)	277(3)
C62	551(8)	6856(7)	2794(6)	130(4)
Cl7	1408(2)	4477(2)	1286(1)	111(1)
Cl8	3214(2)	4620(2)	923(1)	106(1)
Cl9	2276(2)	5889(2)	751(2)	139(1)
C63	2155(6)	4856(5)	747(5)	87(3)

Theoretical Calculations

Computational details

All the DFT calculations except energy decomposition analysis were carried out with Gaussian 09 software package[1]. Geometry optimizations were performed at the M06-2X/6-31G(d) level of theory[2-4]. with frequency calculations employed to ensure that all stationary points were minima. The anisotropy of the induced current density (ACID) calculations were performed at the B3LYP/6-311++G(d,p) level of theory[5-7], using Gaussian 03 software package[8]. The continuous set of gauge transformation (CSGT)[9] method was applied in ACID calculations. The wavefunctions obtained from geometry optimizations were imported to Multiwfn[10] to conduct ELF analyses as well as generate the grid data of reduced density gradient (RDG) and sign(λ_2)ρ functions which are required for the visualization of NCI gradient isosurfaces in VMD software[11]. Based on the number of π electrons in monomer **9**, we selected 31 MOs (Supplementary Table 2) showing π character to separate the ELF_π and ELF_σ. The ADF(2017.104) program[12-13] was used to carry out the energy decomposition analysis at M06-2X/DZP level of theory, with each monomer treated as one fragment. According the evaluations of electron delocalization in monomer **9** (Fig. 4), it could be regarded as a system containing either 38 or 42 π electrons as the phenanthroline moiety could contribute 6 or 10 π electrons to the system depending on an inner or outer ring for the consideration.

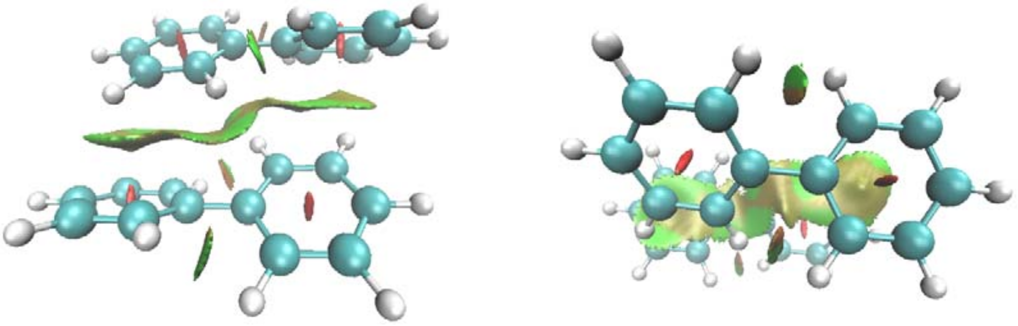

Side view *Top view*

Supplementary Figure 14. Noncovalent interaction between monomers in a diphenyl dimer. An isovalue of 0.70 a.u. is applied to the gradient isosurfaces colored according to values of sign(λ_2)ρ.

Supplementary Table 3. Energy decomposition analysis for the interaction between monomers in catenane **1**. The overall interaction energy (ΔE_{int}) is divided into three terms: ΔE_{elstat} (the quasi-classical electrostatic attraction), ΔE_{Pauli} (the Pauli repulsion), and ΔE_{orb} (the stabilizing orbital interaction). Energies are given in kcal mol^{-1}.

ΔE_{elstat}	ΔE_{Pauli}	ΔE_{orb}	ΔE_{int}
-59.32	12.05	-36.69	-83.97

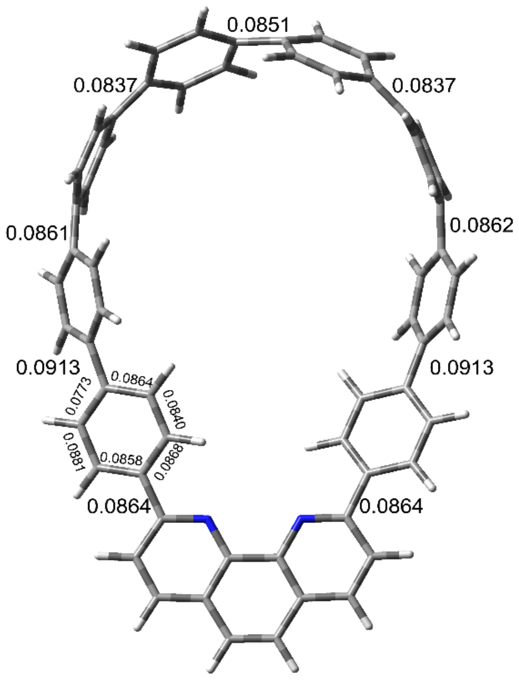

Supplementary Figure 15. The critical isosurface values (CIVs) of ACID for bridging C-C bonds in compound **9**. Larger CIVs indicate stronger delocalization.

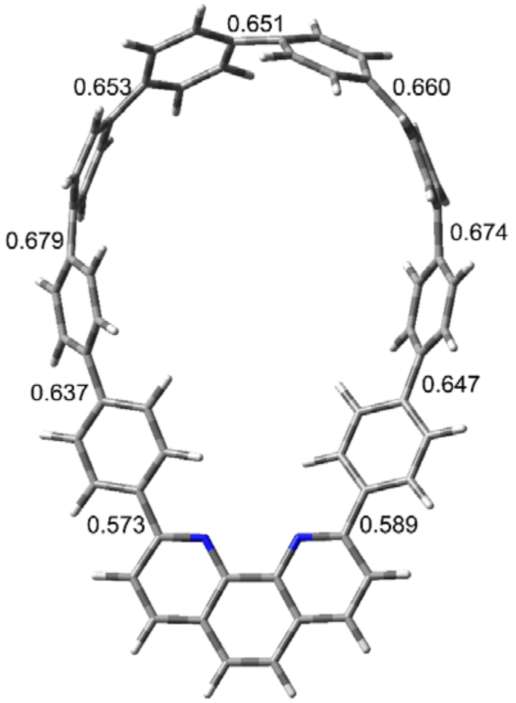

Supplementary Figure 16. ELF_{σ} bifurcation values for the bridging C-C bonds in compound **9**.

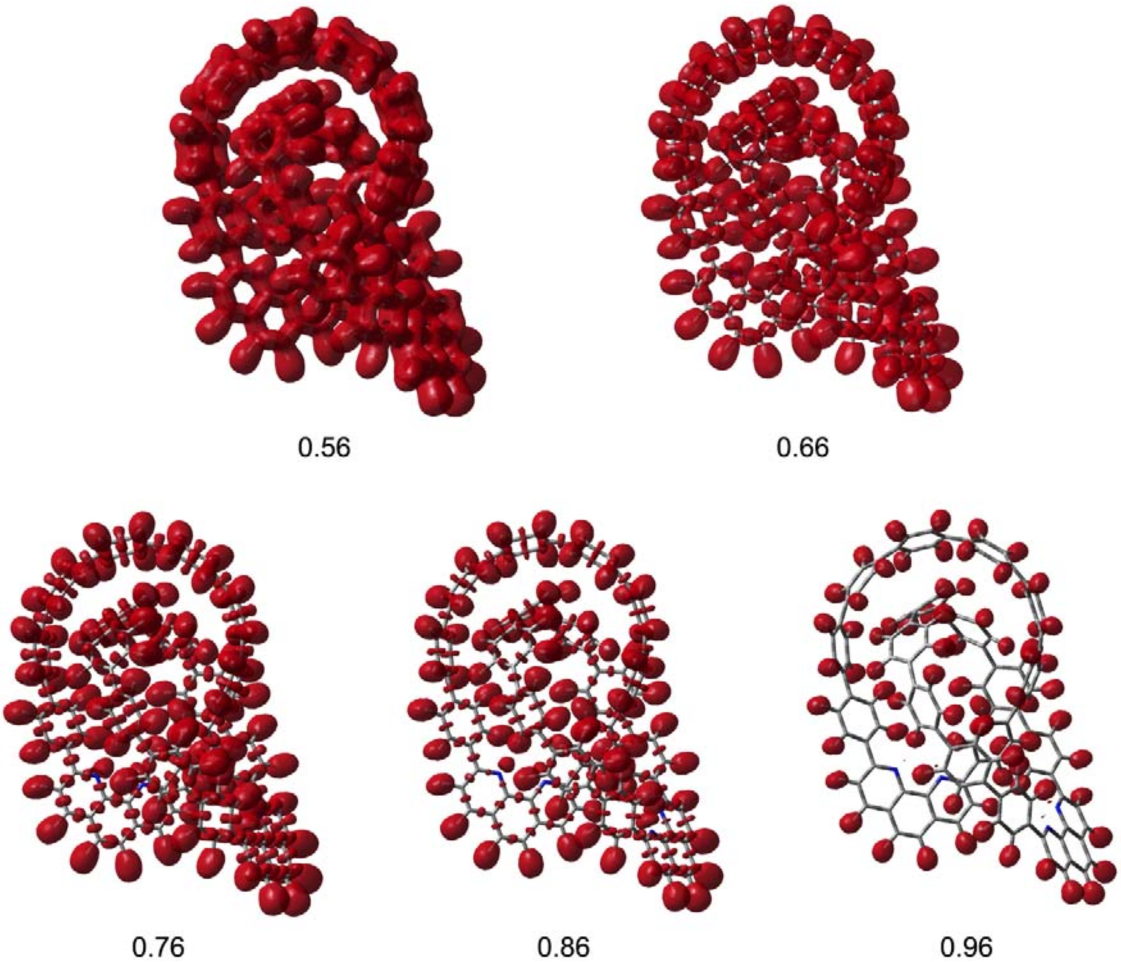

Supplementary Figure 17. ELF_{total} domains in catenane **1** at various isovalues (a.u.). Level of theory: M06-2X/6-31G(d).

Supplementary Table 4. Selected π molecular orbitals for computing the bifurcation values of ELF_{π}. The rest of all occupied molecular orbitals were used to compute the bifurcation values of the ELF_{σ}.

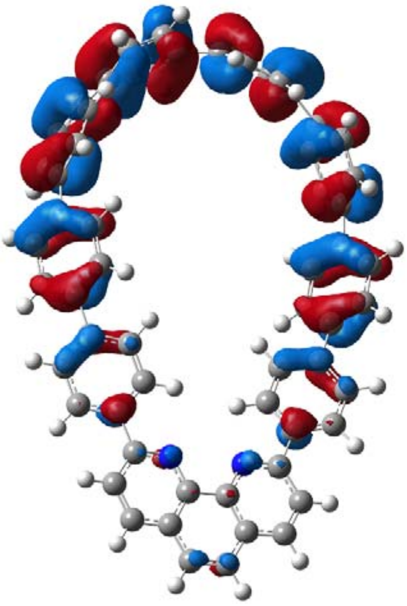 #206 (HOMO)	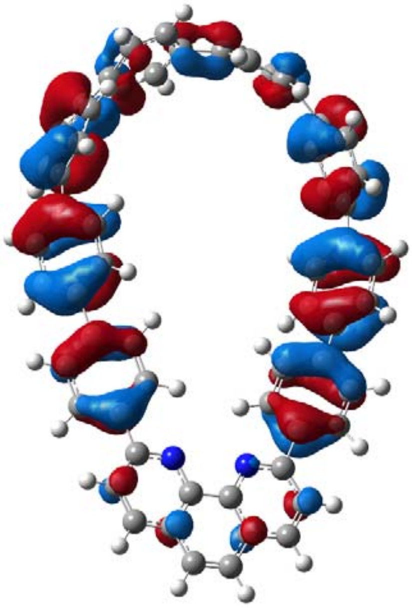 #205	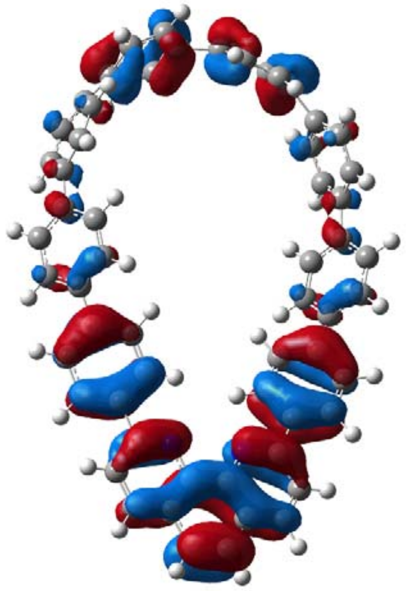 #204
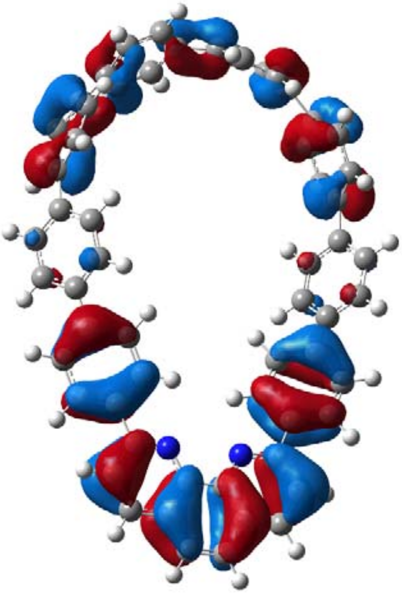 #203	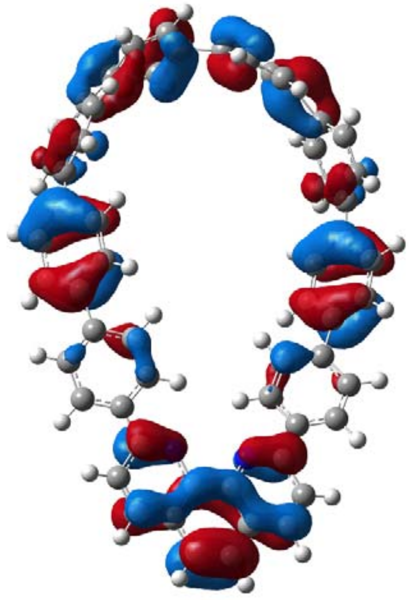 #202	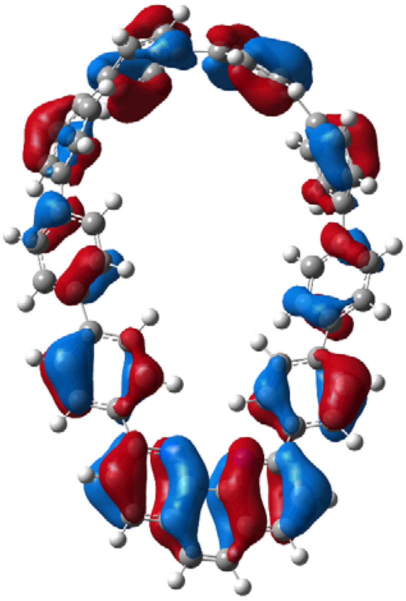 #201

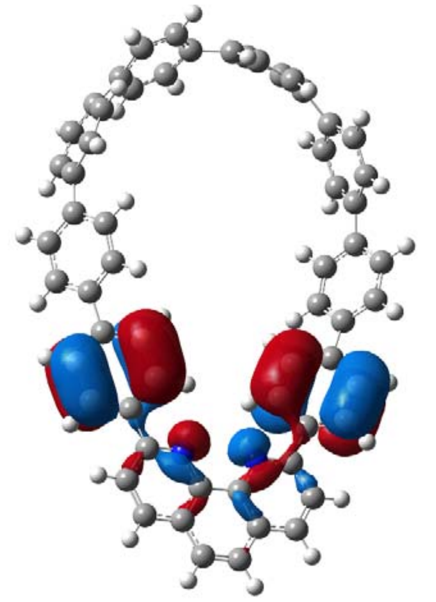

#200

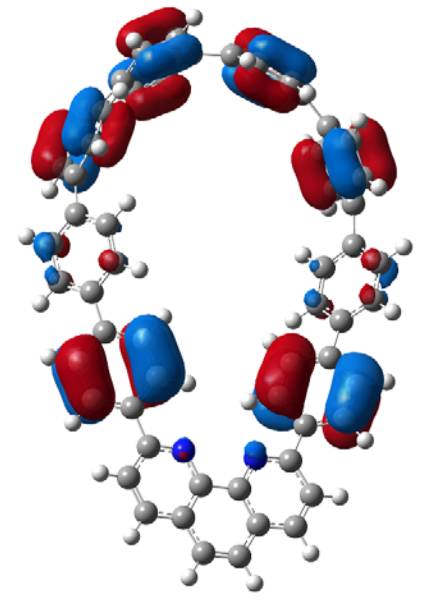

#199

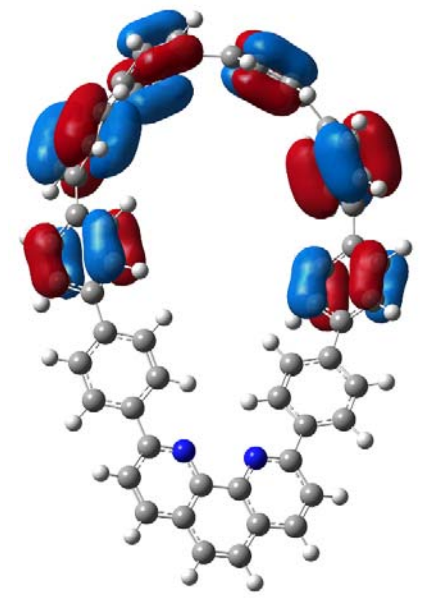

#198

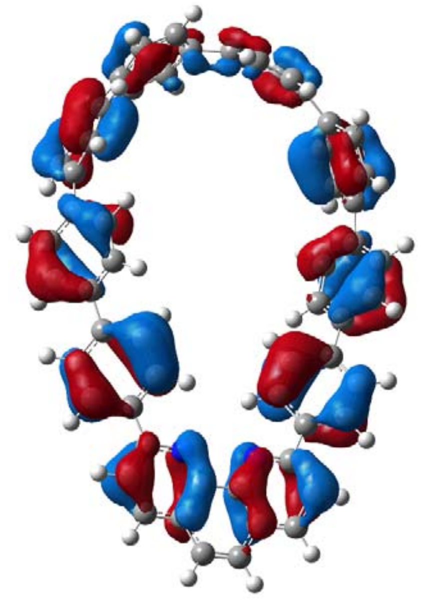

#197

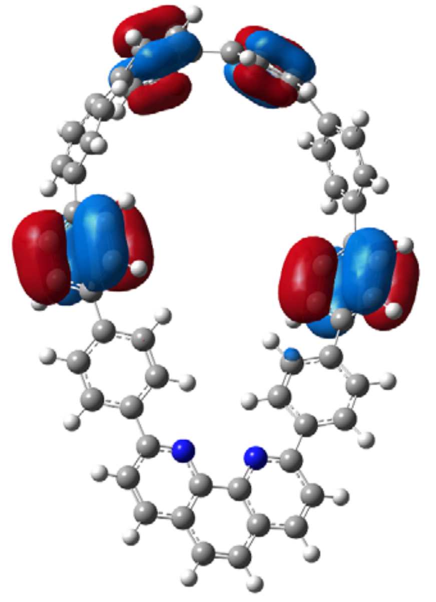

#196

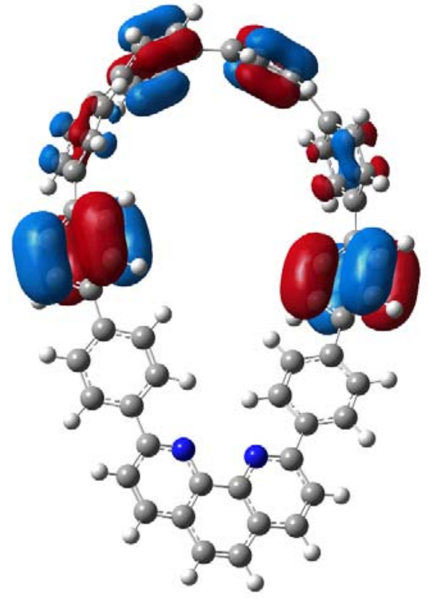

#195

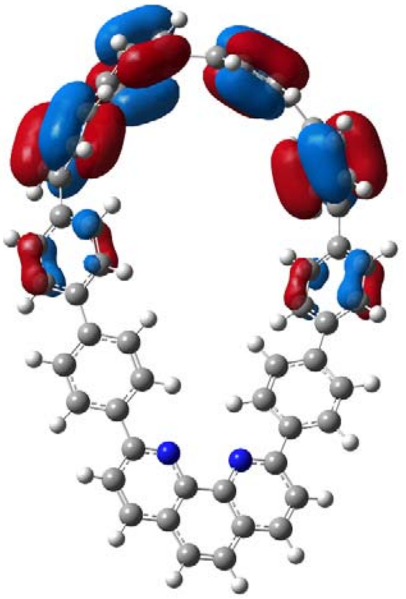

#194

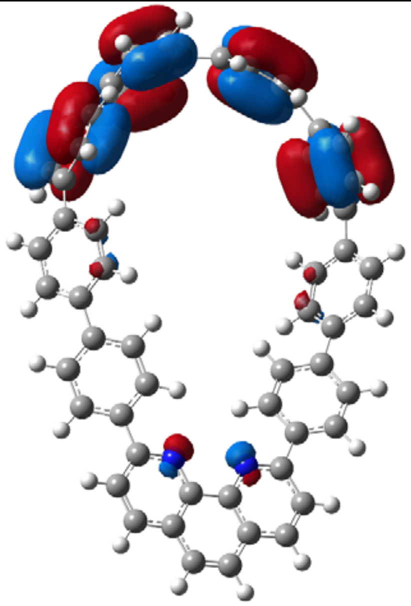

#193

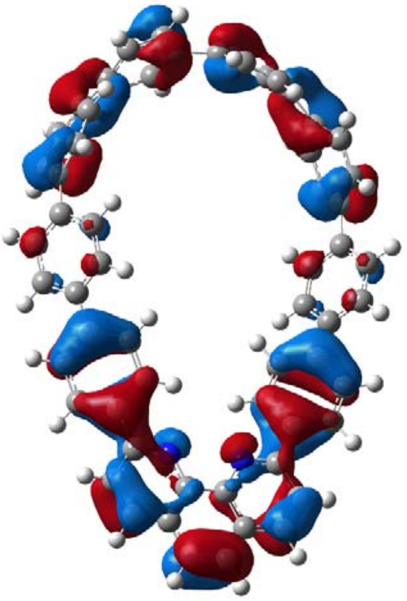

#191

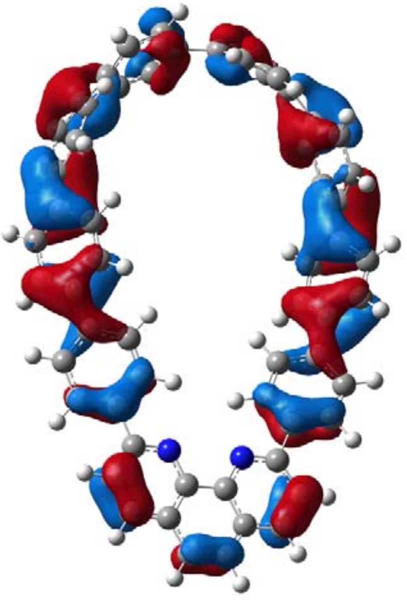

#188

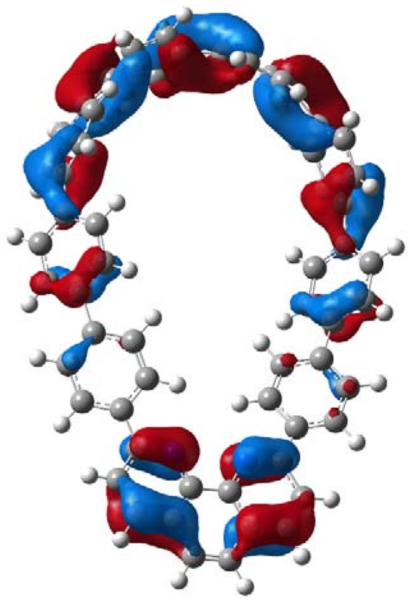

#187

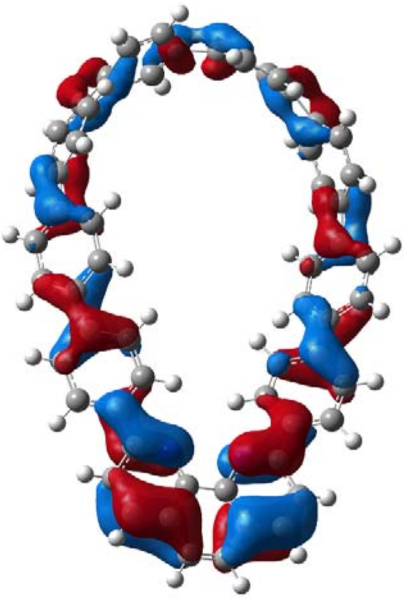

#186

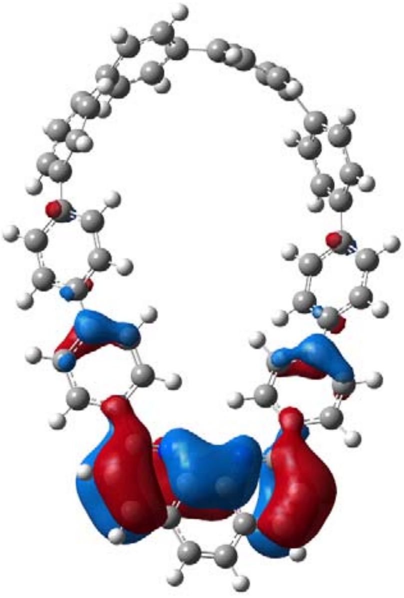

#185

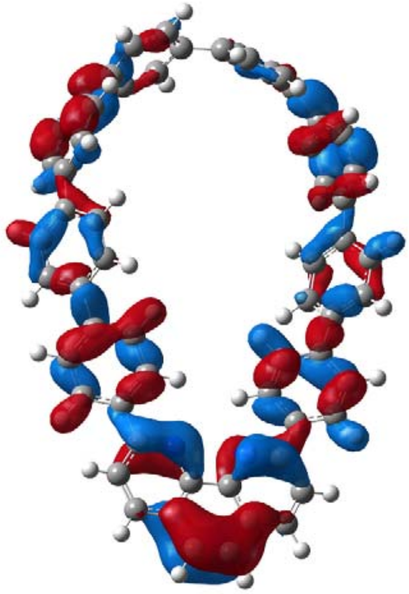

#177

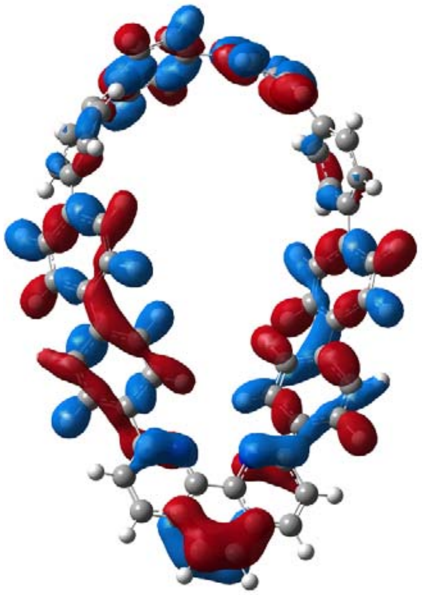

#173

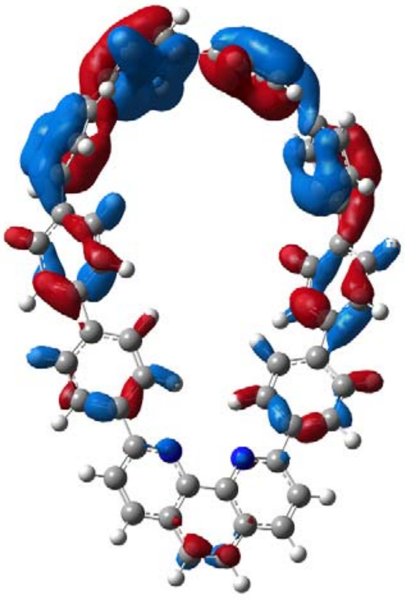

#172

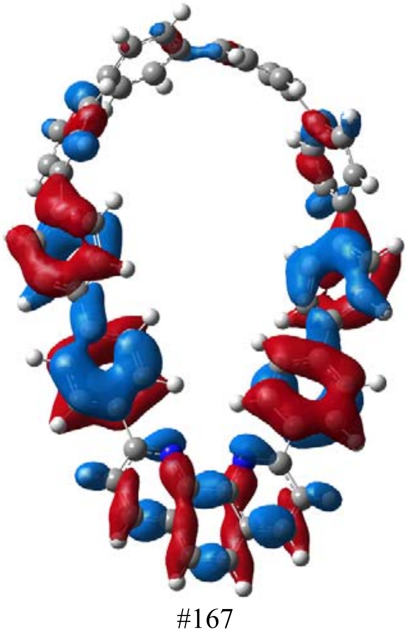

#167

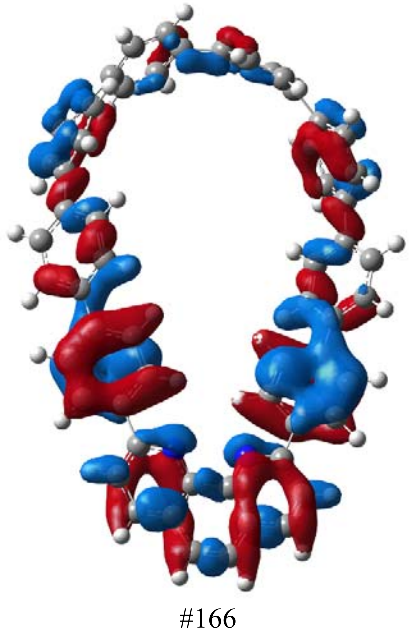

#166

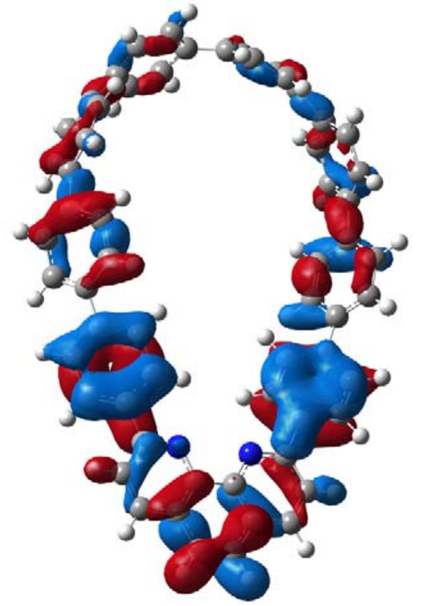

#164

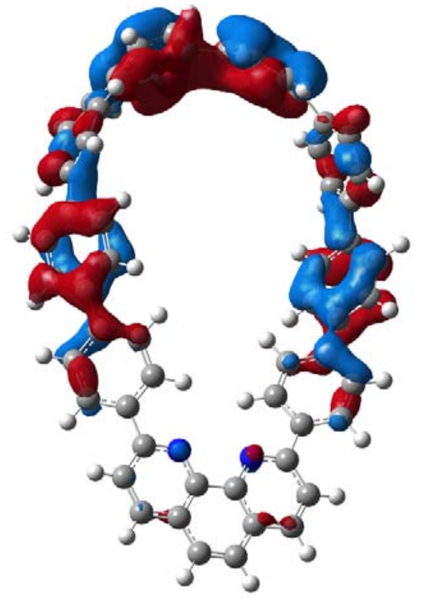

#163

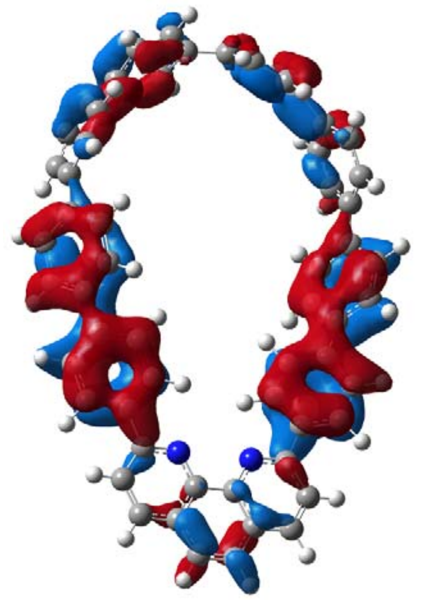

#162

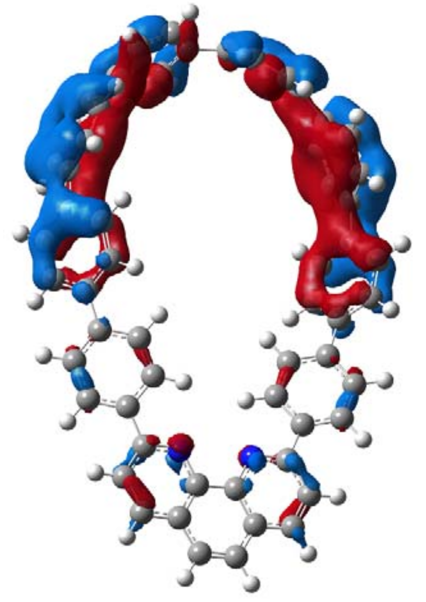

#160

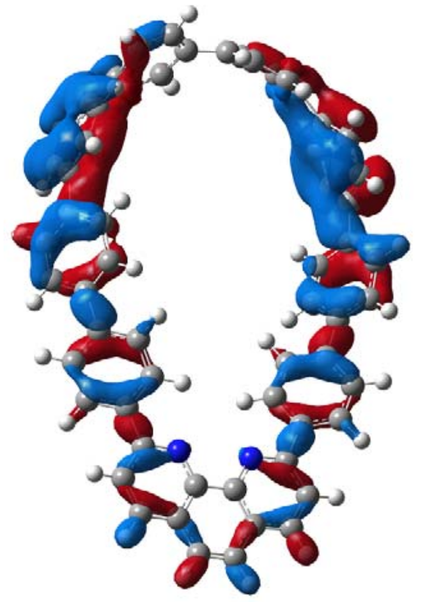

#159

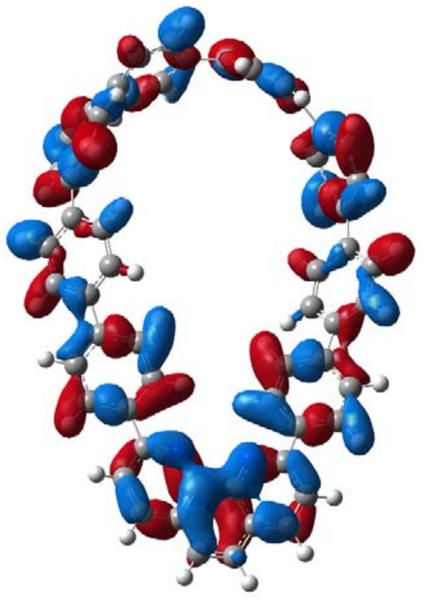

#141

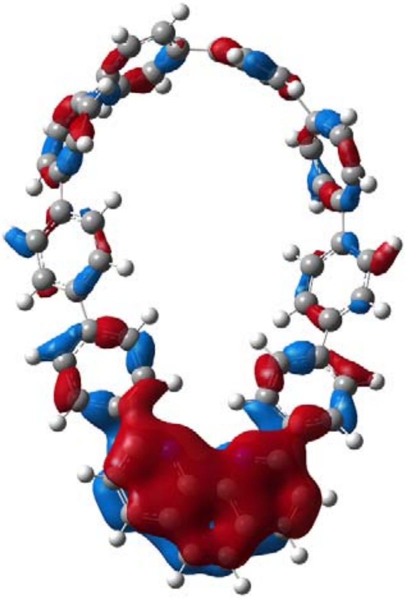 #137		

Supplementary Table 5. Cartesian Coordinates.

Compound	Electronic Energy	Coordinates
**1**	E = -4835.72714509 a.u.	N -8.86780700 -0.98093300 0.55680300
		N -8.26495800 -3.01373700 -1.17958000
		C -9.14421900 0.02267400 1.37560600
		C -10.47181500 0.38006000 1.71556600
		C -11.50839400 -0.35042300 1.18938200
		C -11.23565000 -1.43065000 0.32456200
		C -12.27269700 -2.25104100 -0.23393000
		C -11.97028600 -3.29426200 -1.04558400
		C -10.60690500 -3.59173400 -1.38130900
		C -10.25194600 -4.65699500 -2.23453000
		C -8.93124600 -4.87708900 -2.53780900
		C -7.95117600 -4.01642800 -1.98538500
		C -9.55450800 -2.79796300 -0.86918600
		C -9.87772000 -1.69709200 0.03276400
		C -6.50967900 -4.17198700 -2.30827500
		C -5.94253200 -5.40678400 -2.63765800
		C -4.57979200 -5.51568800 -2.89581600
		C -3.75104000 -4.39006900 -2.84983800
		C -4.32821700 -3.15018900 -2.53913500
		C -5.68223400 -3.04477300 -2.26175200
		C -2.28226900 -4.47543100 -3.03246200
		C -1.53840100 -5.47704300 -2.39658600
		C -0.14991000 -5.42076500 -2.37412600
		C 0.53890600 -4.37088900 -2.99427700
		C -0.20241300 -3.43641100 -3.72537100
		C -1.59042300 -3.48378200 -3.73762100
		C 1.96399100 -4.10224500 -2.68489400
		C 2.32888400 -4.12150300 -1.33652100
		C 3.53733800 -3.59708700 -0.90978100
		C 4.42590700 -3.00789900 -1.81901800

		C	4.11670900	-3.10273600	-3.18453600
		C	2.90389100	-3.63653700	-3.61219300
		C	5.40794400	-2.04309900	-1.26285200
		C	5.88381600	-2.15940700	0.05152000
		C	6.21935500	-1.03118500	0.78680400
		C	6.05750300	0.25517900	0.24368000
		C	5.88634400	0.32978300	-1.14617800
		C	5.56376500	-0.80003300	-1.88668900
		C	5.64980300	1.41627000	1.08640200
		C	5.35869500	2.66775600	0.51758400
		C	4.45443500	3.53803900	1.10942800
		C	3.79749400	3.19569000	2.29861700
		C	4.25653100	2.05860600	2.97384800
		C	5.16379100	1.18962400	2.38185000
		C	2.47792500	3.76904500	2.66161300
		C	1.59629700	4.11591500	1.62861900
		C	0.23067100	4.23241500	1.85468200
		C	-0.29697400	4.00789400	3.13270300
		C	0.60021400	3.82525700	4.19405400
		C	1.96467200	3.70998000	3.96425000
		C	-1.74683600	3.73847400	3.28476100
		C	-2.15018400	2.65306300	4.07612600
		C	-3.44530800	2.16311300	4.01074900
		C	-4.38853600	2.75204300	3.16141500
		C	-4.01917100	3.90253500	2.45649000
		C	-2.71532300	4.38346900	2.50739700
		C	-5.67129700	2.07349300	2.86534300
		C	-5.65611500	0.69320100	2.62195900
		C	-6.78540800	0.03993800	2.15262600
		C	-7.97663100	0.74358700	1.94215800
		C	-8.01191400	2.11192900	2.23340200
		C	-6.87009700	2.77010600	2.67858000
		H	-10.65591400	1.19388700	2.40882000
		H	-12.53967900	-0.11827700	1.44330000
		H	-13.30491000	-2.02102600	0.01598100
		H	-12.75412100	-3.92103300	-1.46218400
		H	-11.03263000	-5.28563600	-2.65543700
		H	-8.64107000	-5.67157300	-3.21664400
		H	-6.55971400	-6.30066000	-2.66062600
		H	-4.15232100	-6.48444800	-3.13983300
		H	-3.69426700	-2.26987000	-2.46889100
		H	-6.12489300	-2.09246400	-1.98825200
		H	-2.05663400	-6.26302100	-1.85239100
		H	0.41311700	-6.17580600	-1.83005100
		H	0.31399400	-2.61464700	-4.21361000
		H	-2.15127200	-2.71704800	-4.26476300
		H	1.59073000	-4.41191900	-0.59663700
		H	3.72263600	-3.51919200	0.15736600
		H	4.80553100	-2.69550100	-3.92017100
		H	2.66421700	-3.64765900	-4.67248100
		H	5.89125100	-3.13123300	0.54211600
		H	6.48723400	-1.16095800	1.83180000
		H	5.81841900	1.29614800	-1.63525300
		H	5.24740900	-0.67074500	-2.91814900
		H	5.75322900	2.93128300	-0.45700300
		H	4.18161200	4.45018800	0.58401500
		H	3.78434200	1.76131900	3.90559000
		H	5.36344800	0.24725400	2.88041200

		H	1.96055000	4.16994400	0.60706700
		H	-0.43733300	4.37560300	1.00533600
		H	0.21621900	3.71990900	5.20537700
		H	2.63263600	3.51194100	4.79840300
		H	-1.41202800	2.13204000	4.67912700
		H	-3.71919900	1.28106300	4.58322400
		H	-4.73757100	4.36980500	1.78600200
		H	-2.43091300	5.22883500	1.88544400
		H	-4.72296400	0.14539900	2.72783400
		H	-6.75095300	-1.01591600	1.90320000
		H	-8.92457100	2.67864800	2.06707900
		H	-6.89954600	3.84063000	2.86371500
		N	-1.70153500	5.37830300	-1.21562300
		N	-3.35140600	3.24679600	-0.61262500
		C	-0.90024000	6.39449800	-1.50274500
		C	-1.39664600	7.69441900	-1.76608200
		C	-2.75255200	7.90159900	-1.74650300
		C	-3.62281900	6.82591900	-1.47968200
		C	-5.04901700	6.97649200	-1.50792500
		C	-5.85881700	5.91232700	-1.28872300
		C	-5.30920200	4.61865500	-1.00046700
		C	-6.12670500	3.49759300	-0.74885600
		C	-5.55248200	2.29244100	-0.43277000
		C	-4.14002500	2.21161600	-0.35467400
		C	-3.90739900	4.42785000	-0.93296100
		C	-3.03423200	5.56730000	-1.20786700
		C	-3.48213900	0.96793300	0.11758200
		C	-4.03416800	-0.30361900	-0.08070500
		C	-3.40599200	-1.43950400	0.42167300
		C	-2.21204300	-1.33307700	1.14751000
		C	-1.67177200	-0.05396200	1.34909800
		C	-2.29025000	1.07694200	0.83896200
		C	-1.46370100	-2.51362700	1.64396900
		C	-0.73401200	-2.42913900	2.83534200
		C	0.17766700	-3.41072100	3.19002100
		C	0.38917200	-4.53803000	2.38334000
		C	-0.44729000	-4.68728600	1.26577100
		C	-1.34615500	-3.69152100	0.89488200
		C	1.63464400	-5.33166700	2.57199700
		C	2.39453200	-5.25283600	3.75108200
		C	3.75680800	-5.51616400	3.74599900
		C	4.42241700	-5.85842900	2.55828300
		C	3.62058200	-6.18073300	1.45745500
		C	2.25212000	-5.94249600	1.47424400
		C	5.86709500	-5.57954100	2.35481400
		C	6.46078200	-4.53680700	3.07920900
		C	7.62173700	-3.92130300	2.63274300
		C	8.23135400	-4.31105100	1.43323200
		C	7.74756900	-5.47312900	0.81554800
		C	6.59198000	-6.09744400	1.26996800
		C	9.06956700	-3.31286700	0.72306000
		C	8.81949000	-3.09660300	-0.63654100
		C	9.12790200	-1.88257400	-1.23485000
		C	9.71027800	-0.84676700	-0.49764800
		C	10.14490900	-1.13950700	0.80407100
		C	9.82701200	-2.34969100	1.40533200
		C	9.56191000	0.54492300	-0.98781400
		C	9.47366500	0.85420500	-2.35298200

		C	8.87020900	2.02962000	-2.77969800
		C	8.34449800	2.94379600	-1.85539200
		C	8.60771000	2.72079200	-0.49857700
		C	9.20120100	1.54040300	-0.07352600
		C	7.26377300	3.89913500	-2.20604900
		C	6.98689700	5.03719500	-1.43631900
		C	5.72266400	5.61742400	-1.44970800
		C	4.69342800	5.08151600	-2.23505600
		C	5.02407100	4.05734700	-3.13107900
		C	6.28518900	3.47944300	-3.11854200
		C	3.27530700	5.46708800	-2.04024900
		C	2.28602700	4.47256300	-2.09279700
		C	0.94821200	4.77656500	-1.88953100
		C	0.55064400	6.09207700	-1.62369500
		C	1.53714400	7.08110300	-1.53504900
		C	2.87814800	6.77405000	-1.73807700
		H	-0.71892700	8.50144200	-2.01938500
		H	-3.16908900	8.88249400	-1.96132400
		H	-5.46121800	7.95890000	-1.72155700
		H	-6.93992300	6.01778800	-1.32152200
		H	-7.20831000	3.60474400	-0.77952800
		H	-6.16733400	1.43215000	-0.18499000
		H	-4.96727900	-0.41404900	-0.62826900
		H	-3.85505800	-2.41566400	0.25594800
		H	-0.73092700	0.05394200	1.88081500
		H	-1.85805800	2.06135200	0.99193600
		H	-0.84170300	-1.55072900	3.46517100
		H	0.79822700	-3.24637000	4.06434200
		H	-0.35635200	-5.56268000	0.62776300
		H	-1.90344900	-3.79997400	-0.03348500
		H	1.93358700	-4.90603000	4.67083400
		H	4.32405900	-5.37069600	4.66091500
		H	4.07794200	-6.47728300	0.51832200
		H	1.69586900	-6.10609900	0.55851400
		H	5.93787300	-4.10406200	3.92607900
		H	7.97217800	-3.02917100	3.14375900
		H	8.23732800	-5.84840900	-0.07889300
		H	6.20938000	-6.95677900	0.72685800
		H	8.21962300	-3.81260600	-1.19076200
		H	8.75894200	-1.68757600	-2.23721500
		H	10.67359000	-0.38257700	1.37696800
		H	10.10390900	-2.51927100	2.44259200
		H	9.81470800	0.13253600	-3.09008100
		H	8.74464700	2.20939800	-3.84423000
		H	8.20341600	3.39942200	0.24670800
		H	9.23980300	1.33130400	0.99130600
		H	7.73943100	5.41612300	-0.75050900
		H	5.50570700	6.43388500	-0.76563500
		H	4.26323700	3.66730800	-3.80080600
		H	6.47579800	2.61089300	-3.74301400
		H	2.57808500	3.44052900	-2.26515600
		H	0.18848500	4.00215400	-1.92672900
		H	1.26489500	8.10453000	-1.29502000
		H	3.62317100	7.56293300	-1.68203200
**9**	E = -2417.83349459 a.u.	C	4.63150700	0.36563300	4.71120500
		C	5.15436700	0.30420400	3.42172600
		H	6.18860000	0.58844900	3.24708400
		C	4.36938800	-0.13692500	2.35178400

		C	3.04024900	-0.49935000	2.61565400
		H	2.40110900	-0.80694300	1.79359200
		C	2.51635500	-0.43272200	3.89421100
		H	1.48213700	-0.69592300	4.08730200
		C	3.30671900	-0.00414500	4.96715000
		C	-4.63150700	-0.36563300	4.71120500
		C	-3.30671900	0.00414500	4.96715000
		C	-2.51635500	0.43272200	3.89421100
		H	-1.48213700	0.69592300	4.08730200
		C	-3.04024900	0.49935000	2.61565400
		H	-2.40110900	0.80694300	1.79359200
		C	-4.36938800	0.13692500	2.35178400
		C	-5.15436700	-0.30420400	3.42172600
		H	-6.18860000	-0.58844900	3.24708400
		C	-4.85353600	0.19701900	0.95161100
		C	-4.41475300	1.23213300	0.11806900
		H	-3.83779100	2.04913100	0.54115900
		C	-4.65607200	1.20292000	-1.24494100
		H	-4.21882800	1.97409300	-1.87044700
		C	-5.36036800	0.14542200	-1.83751200
		C	-5.89401700	-0.83259900	-0.98819700
		H	-6.47594300	-1.64927700	-1.40661400
		C	-5.64279000	-0.80880800	0.38106600
		H	-6.00743700	-1.62027600	1.00533600
		C	-5.31137700	0.00984300	-3.31511500
		C	-5.18183200	-1.24860700	-3.91666100
		H	-5.34769300	-2.14593900	-3.32751300
		C	-4.70341200	-1.37768200	-5.21464300
		H	-4.54936400	-2.37445400	-5.61658700
		C	-4.29982300	-0.25522800	-5.95192400
		C	-4.62202000	1.00127300	-5.41973200
		H	-4.36181500	1.90262300	-5.96515500
		C	-5.13195100	1.13031200	-4.13639700
		H	-5.30399900	2.12480200	-3.73557700
		C	-3.28721100	-0.34947800	-7.03807200
		C	-2.95816400	0.74417100	-7.85525200
		H	-3.65873400	1.56662000	-7.96686000
		C	-1.69875200	0.85340700	-8.43252300
		H	-1.43397200	1.76246000	-8.96639100
		C	-0.72955600	-0.13941700	-8.23264400
		C	-1.14715900	-1.33209600	-7.63076500
		H	-0.42593800	-2.12774200	-7.46920100
		C	-2.39785800	-1.43171400	-7.03911300
		H	-2.60902800	-2.30764800	-6.43524000
		C	0.72955600	0.13941700	-8.23264400
		C	1.69875200	-0.85340700	-8.43252300
		H	1.43397200	-1.76246000	-8.96639100
		C	2.95816400	-0.74417100	-7.85525200
		H	3.65873400	-1.56662000	-7.96686000
		C	3.28721100	0.34947800	-7.03807200
		C	2.39785800	1.43171400	-7.03911300
		H	2.60902800	2.30764800	-6.43524000
		C	1.14715900	1.33209600	-7.63076500
		H	0.42593800	2.12774200	-7.46920100
		C	4.29982300	0.25522800	-5.95192400
		C	4.62202000	-1.00127300	-5.41973200
		H	4.36181500	-1.90262300	-5.96515500
		C	5.13195100	-1.13031200	-4.13639700

		H	5.30399900	-2.12480200	-3.73557700
		C	5.31137700	-0.00984300	-3.31511500
		C	5.18183200	1.24860700	-3.91666100
		H	5.34769300	2.14593900	-3.32751300
		C	4.70341200	1.37768200	-5.21464300
		H	4.54936400	2.37445400	-5.61658700
		C	5.36036800	-0.14542200	-1.83751200
		C	5.89401700	0.83259900	-0.98819700
		H	6.47594300	1.64927700	-1.40661400
		C	5.64279000	0.80880800	0.38106600
		H	6.00743700	1.62027600	1.00533600
		C	4.85353600	-0.19701900	0.95161100
		C	4.41475300	-1.23213300	0.11806900
		H	3.83779100	-2.04913100	0.54115900
		C	4.65607200	-1.20292000	-1.24494100
		H	4.21882800	-1.97409300	-1.87044700
		H	5.26563800	0.72496800	5.51656000
		H	-5.26563800	-0.72496800	5.51656000
		C	-3.46558300	-0.07022000	7.50595100
		C	-2.82222400	-0.07267900	8.71971100
		C	-1.41298500	-0.04234500	8.76976100
		C	-0.72955600	-0.01699400	7.53280700
		C	-2.69063300	-0.04159100	6.32029800
		C	-0.67799500	-0.02238100	10.00288600
		C	0.72955600	0.01699400	7.53280700
		C	1.41298500	0.04234500	8.76976100
		C	0.67799500	0.02238100	10.00288600
		C	2.82222400	0.07267900	8.71971100
		H	3.38823000	0.08369800	9.64782200
		C	3.46558300	0.07022000	7.50595100
		C	2.69063300	0.04159100	6.32029800
		H	-1.23354400	-0.04021100	10.93663500
		H	-4.54869900	-0.06163800	7.45680000
		H	-3.38823000	-0.08369800	9.64782200
		H	1.23354400	0.04021100	10.93663500
		H	4.54869900	0.06163800	7.45680000
		N	1.36687300	0.01915100	6.35213100
		N	-1.36687300	-0.01915100	6.35213100
**Diphenyl dimer**	E = -926.208778914 a.u.	C	-4.56508000	0.23756600	-0.13186700
		C	-3.74527300	0.32264200	-1.25498600
		C	-2.55801800	-0.39923300	-1.31230000
		C	-2.16346600	-1.21704700	-0.24710100
		C	-2.99308600	-1.29000000	0.87887900
		C	-4.18491100	-0.57306700	0.93546300
		C	-0.88273600	-1.96645800	-0.29136600
		C	0.25735000	-1.39540600	-0.86991600
		C	1.47328500	-2.07076600	-0.86631900
		C	1.56632400	-3.33763200	-0.29621000
		C	0.43543000	-3.92471400	0.26731000
		C	-0.77737500	-3.24372200	0.27261300
		H	-4.03312900	0.95056100	-2.09262000
		H	-1.93217500	-0.34081800	-2.19810600
		H	-2.68737000	-1.89704100	1.72666400
		H	-4.81244700	-0.64076000	1.81917800
		H	0.20152700	-0.39226400	-1.28459400
		H	2.35291600	-1.59051400	-1.28605300
		H	0.49654400	-4.91726700	0.70355900
		H	-1.65959300	-3.71288600	0.69946900

		C	-1.56661700	3.33709800	0.29687900
		C	-0.43592500	3.92439000	-0.26682500
		C	0.77699000	3.24359200	-0.27238700
		C	0.88265900	1.96631600	0.29150300
		C	-0.25722500	1.39504400	0.87023000
		C	-1.47326600	2.07021200	0.86689400
		C	2.16350600	1.21712400	0.24696700
		C	2.99283200	1.29020400	-0.87922900
		C	4.18479400	0.57353300	-0.93607500
		C	4.56540900	-0.23695900	0.13121100
		C	3.74589500	-0.32217400	1.25452900
		C	2.55848100	0.39943700	1.31210000
		H	-0.49727700	4.91695800	-0.70300700
		H	1.65906000	3.71292100	-0.69937100
		H	-0.20116000	0.39188300	1.28483500
		H	-2.35273400	1.58976000	1.28673600
		H	2.68677700	1.89717500	-1.72694100
		H	4.81211800	0.64133000	-1.81993100
		H	4.03413900	-0.94997700	2.09211600
		H	1.93288300	0.34094800	2.19807500
		H	-2.51686500	3.86226900	0.28861500
		H	5.49356300	-0.79840300	0.08733300
		H	-5.49311600	0.79922100	-0.08819700
		H	2.51647700	-3.86297000	-0.28775600

Optoelectronic Properties

UV-Vis absorbance spectra were recorded in a 1 cm quartz cuvette using a Hitachi U-3900 spectrophotometer. The extinction coefficients were calculated based on Beer-Lambert plots. Steady-state emission spectra were recorded using a Hitachi F-4500 spectrofluorimeter. Time-resolved emission data were collected by the time correlate single photon counting (TCSPC) capability on an Edinburgh FLS-920 instrument. The pulsed excitation light (340 nm) was generated by an Edinburgh EPL-340 ps pulsed laser diode. The absolute photoluminescence quantum yield was measured using an integrating sphere coupled with an Edinburgh FLS-920 instrument.

Cyclic voltammetry experiments were performed using a Princeton Applied Research Potentionstat-gravanostat Model-283. Measurements were carried out in degassed 0.1 M *n*-Bu_4NPF_6 solutions in THF under an argon atmosphere, with a glassy carbon disc working electrode, a platinum wire counter electrode, and a saturated calomel (SCE) reference electrode. Ferrocene/ferrocenium couple was used as an internal reference (Connelly, N. G.; Geiger, W. E. *Chem. Rev.* **1996**, *96*, 877). Due to the poor solubility, cyclic voltammetry characterization of compound **9** was unsuccessful.

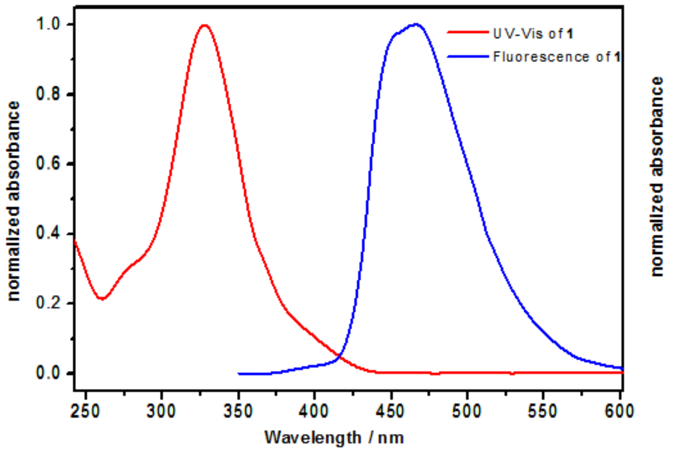

Supplementary Figure 18. UV-Vis and fluorescence spectra of compound **1** in CH_2Cl_2 (2.5×10^{-6} M) at 25 °C. $\lambda_{max} = 327$ nm, $\varepsilon = 5.44 \times 10^5$ L·mol^{-1}·cm^{-1}; excitation wavelength 300 nm, maximum emission wavelength 466 nm.

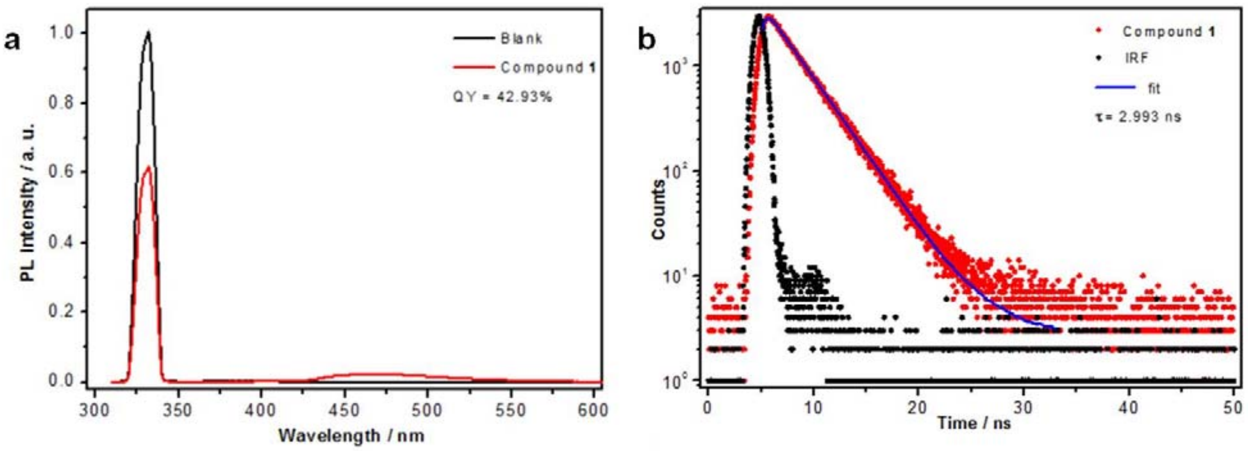

Supplementary Figure 19. a, Quantum yield and **b,** emission lifetime of compound **1** in CH_2Cl_2.

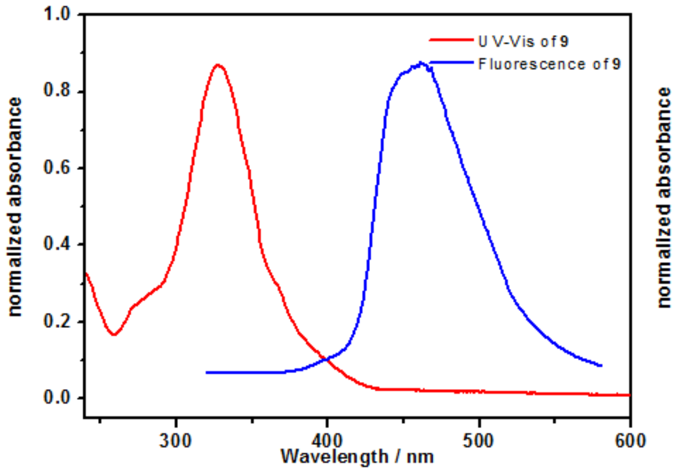

Supplementary Figure 20. UV-Vis and fluorescence spectra of compound **9** in CH_2Cl_2 (1.8×10^{-6} M) at 25 °C . λ_{max} = 327 nm, $\varepsilon = 4.77 \times 10^5$ $L \cdot mol^{-1} \cdot cm^{-1}$; excitation wavelength 300 nm, maximum emission wavelength 465 nm.

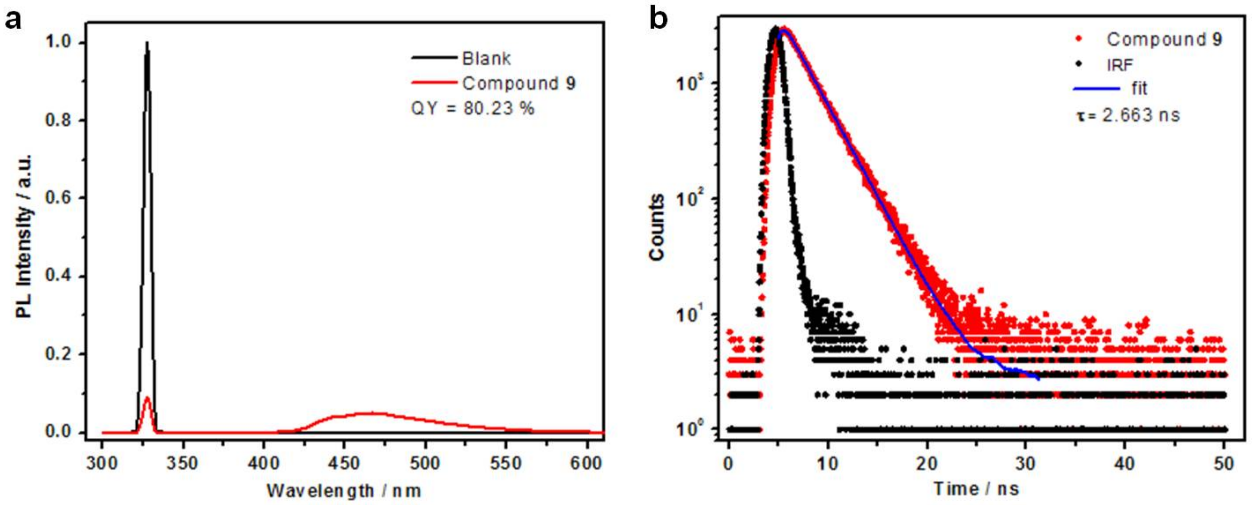

Supplementary Figure 21. a, Quantum yield and **b,** emission lifetime of compound **9** in CH_2Cl_2**.**

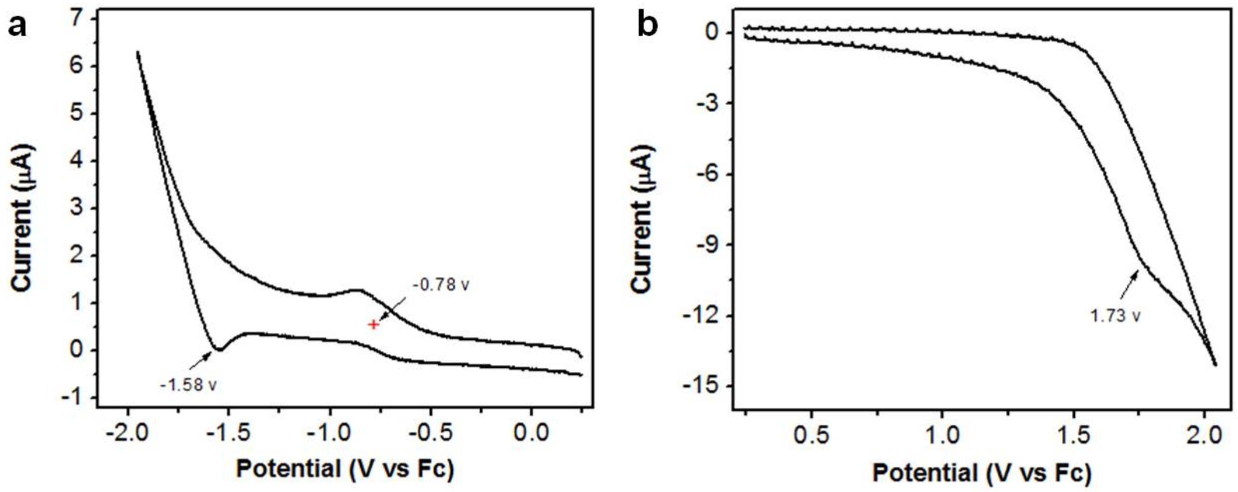

Supplementary Figure 22. a, Reductive and **b,** oxidative potentials of compound **1** (*vs*. ferrocene$^{+1/0}$, scan rate = 100 mV s^{-1}).

Supplementary NMR Spectra

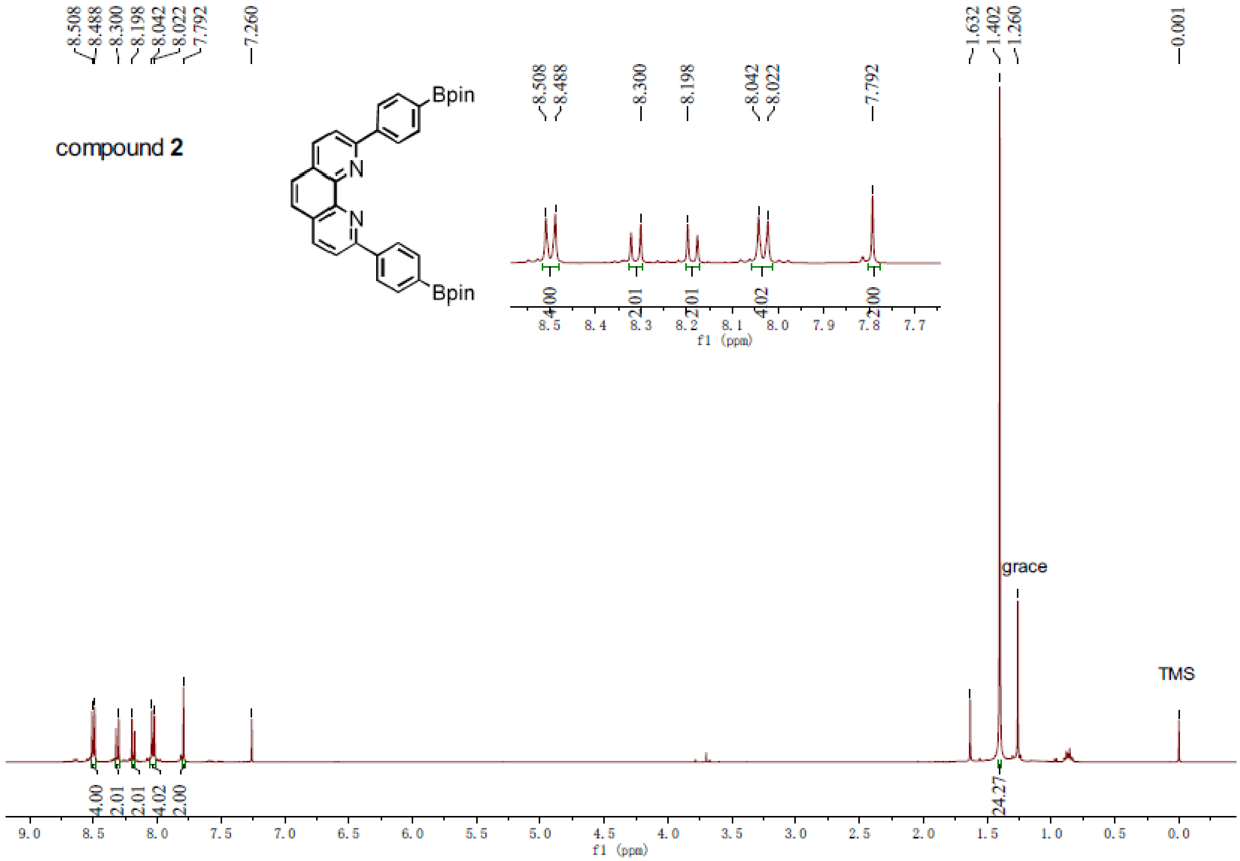

Supplementary Figure 23. ^{1}H NMR spectra for compound **2**.

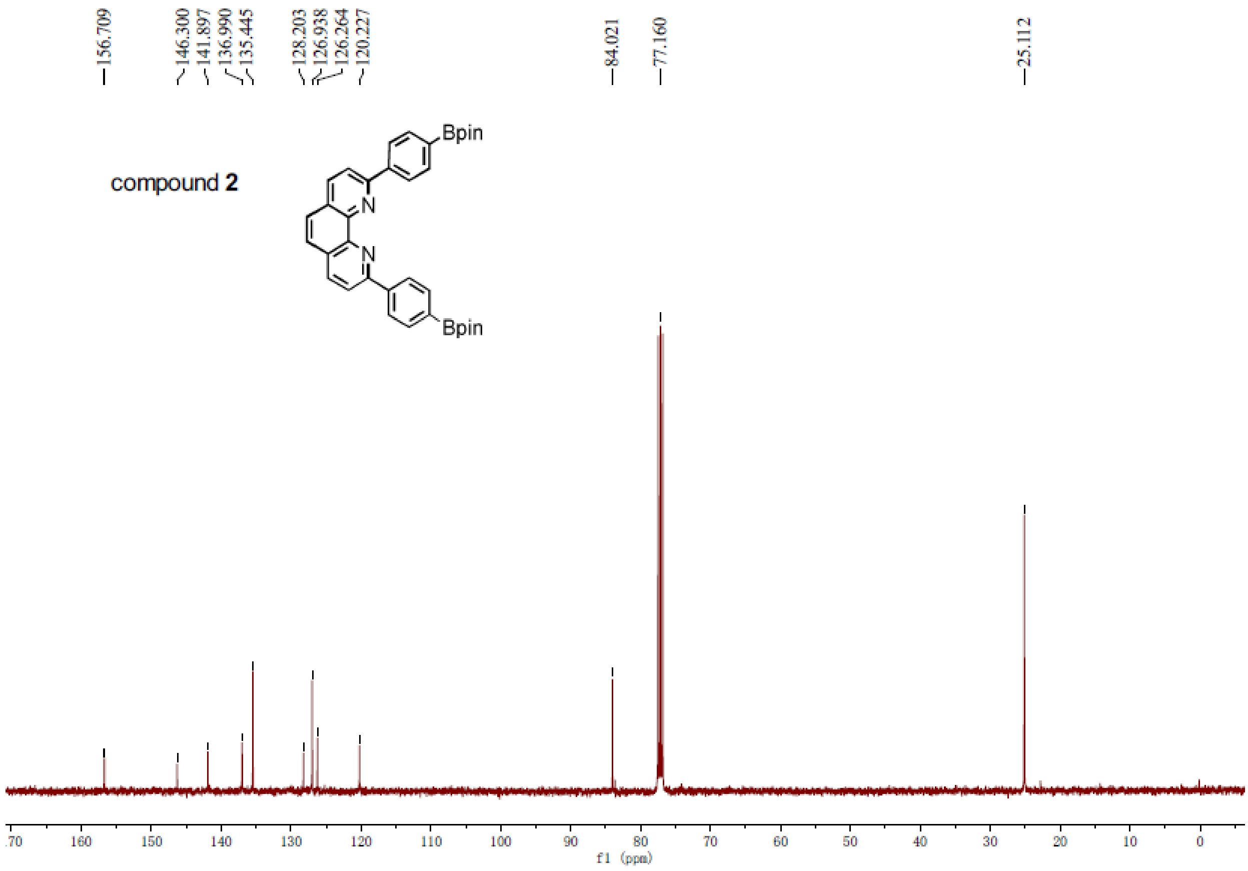

Supplementary Figure 24. ^{13}C NMR spectra for compound **2**.

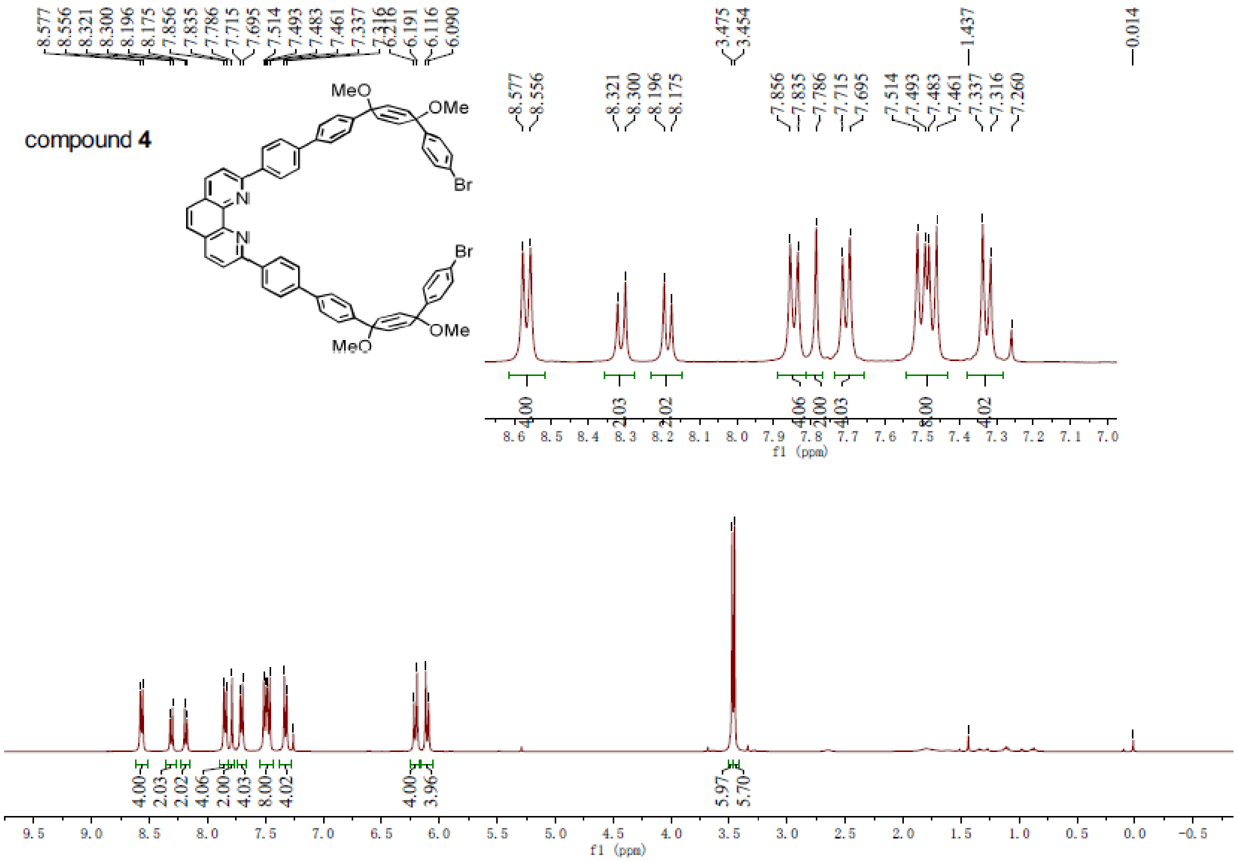

Supplementary Figure 25. ^{1}H NMR spectra for compound **4**.

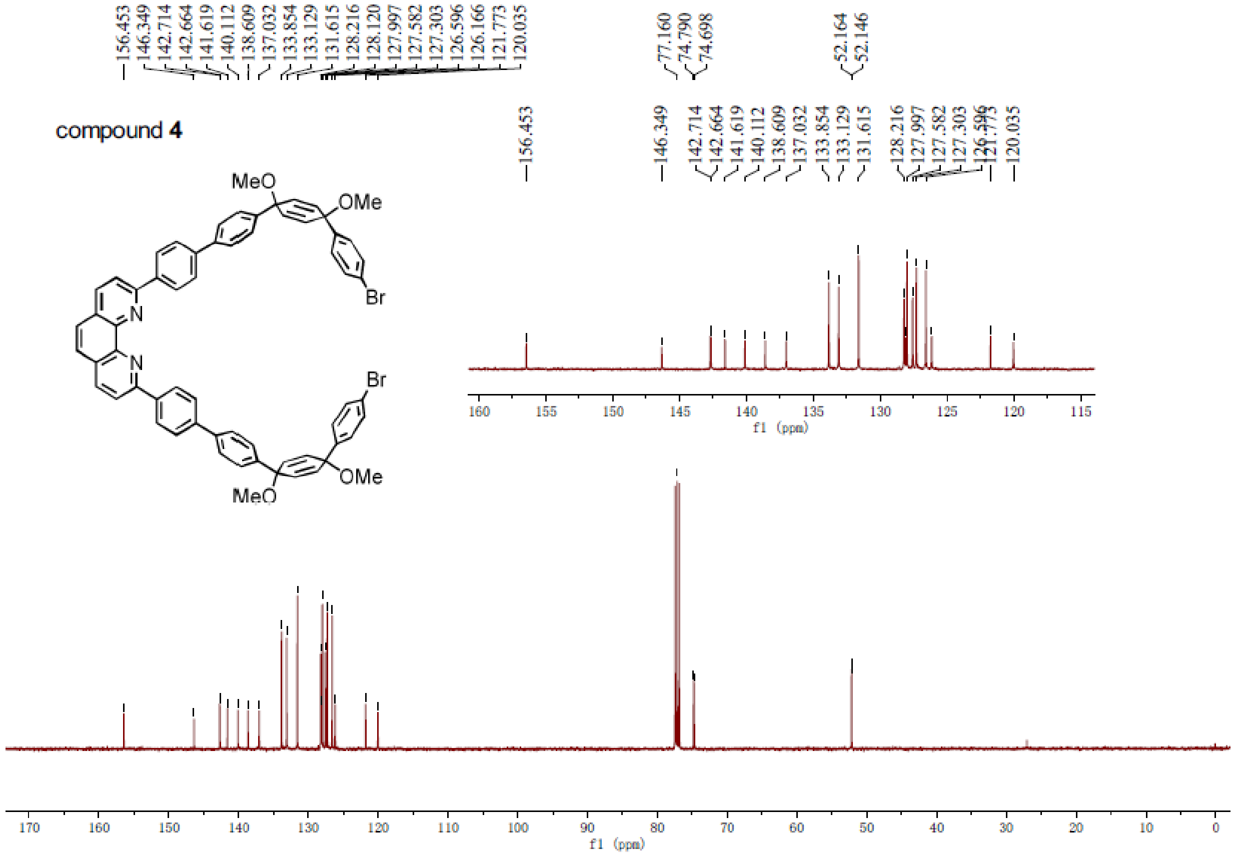

Supplementary Figure 26. ^{13}C NMR spectra for compound **4**.

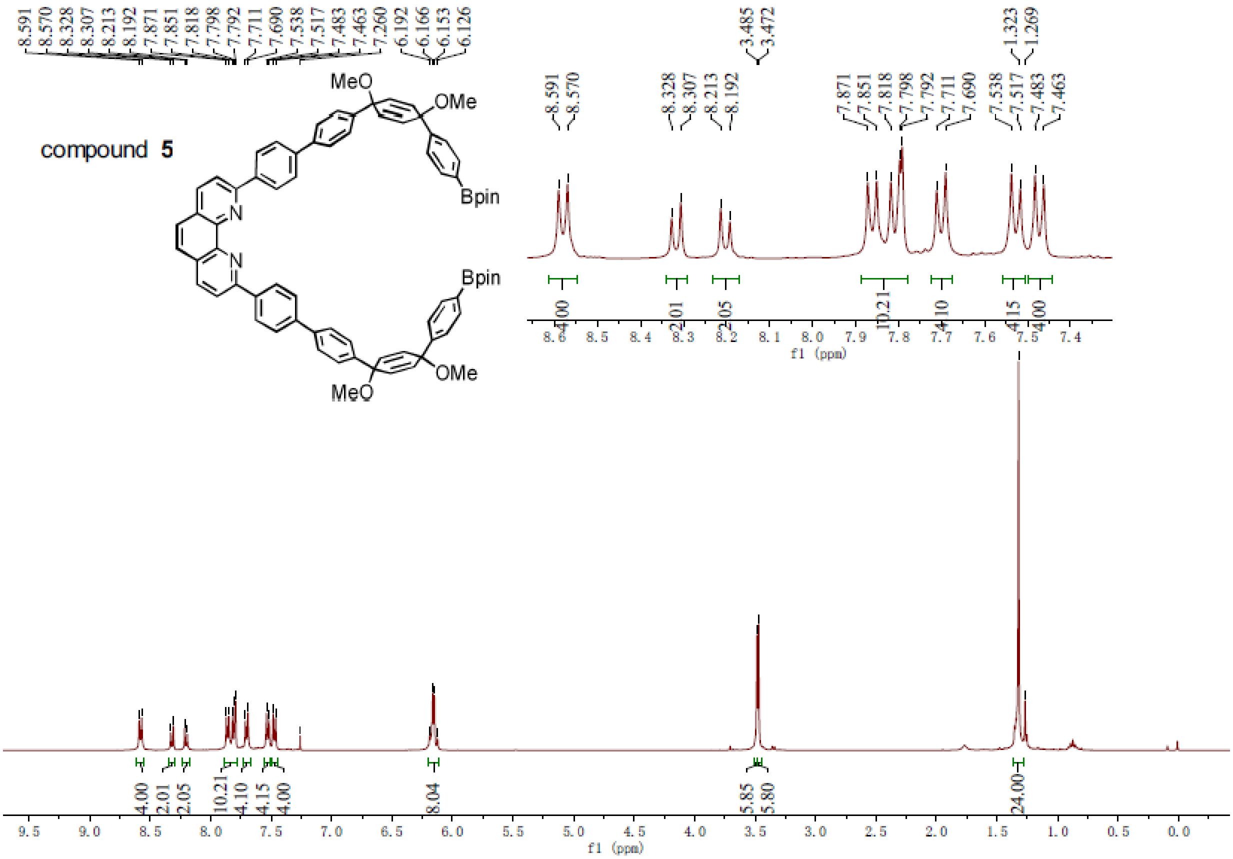

Supplementary Figure 27. ^{1}H NMR spectra for compound **5**.

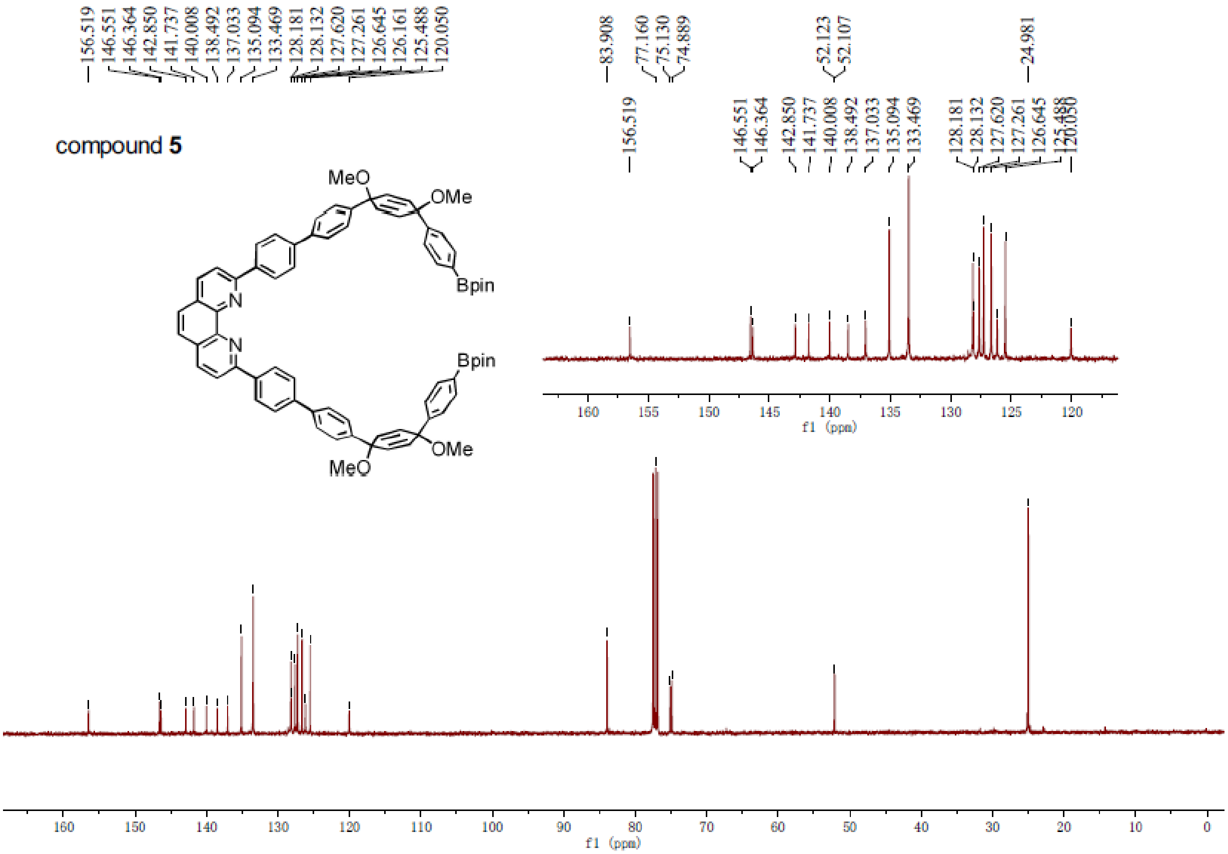

Supplementary Figure 28. ^{13}C NMR spectra for compound **5**.

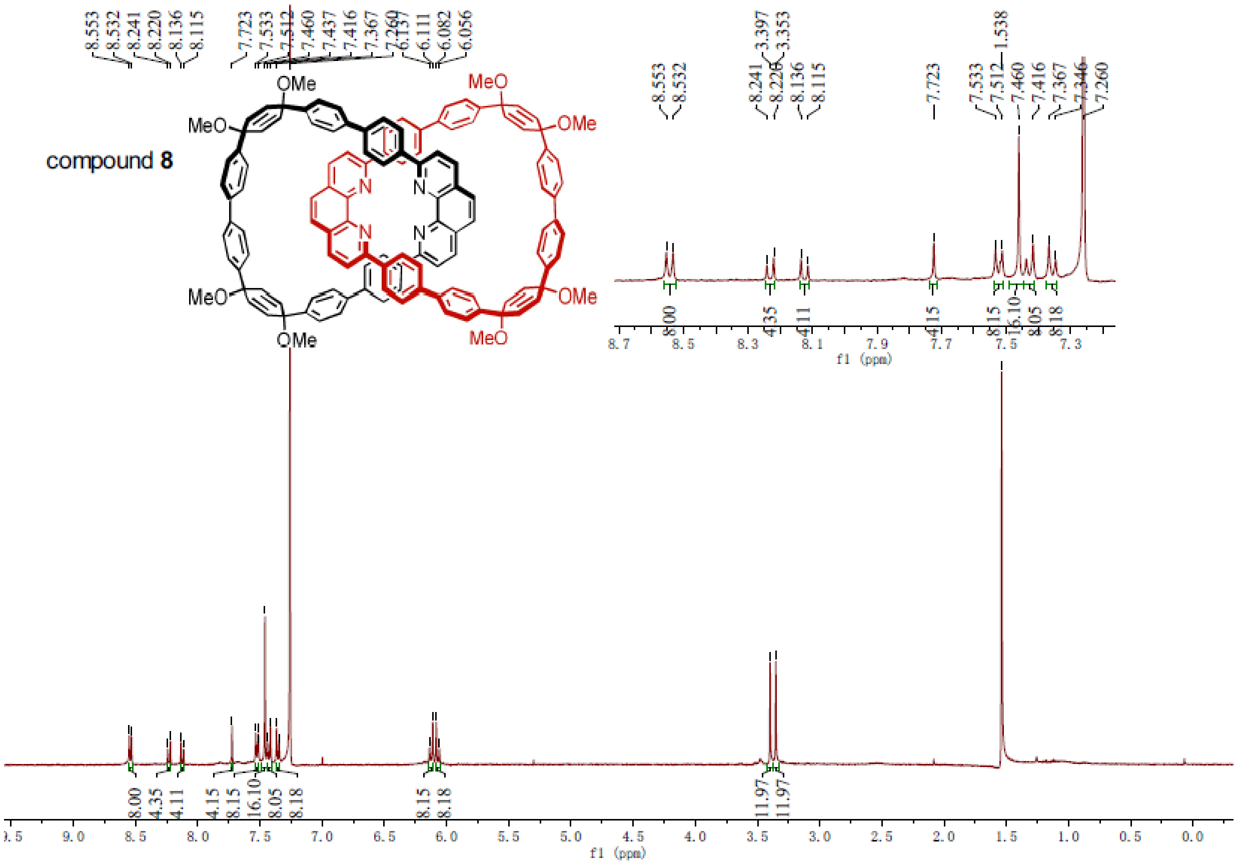

Supplementary Figure 29. ^{1}H NMR spectra for compound **8**.

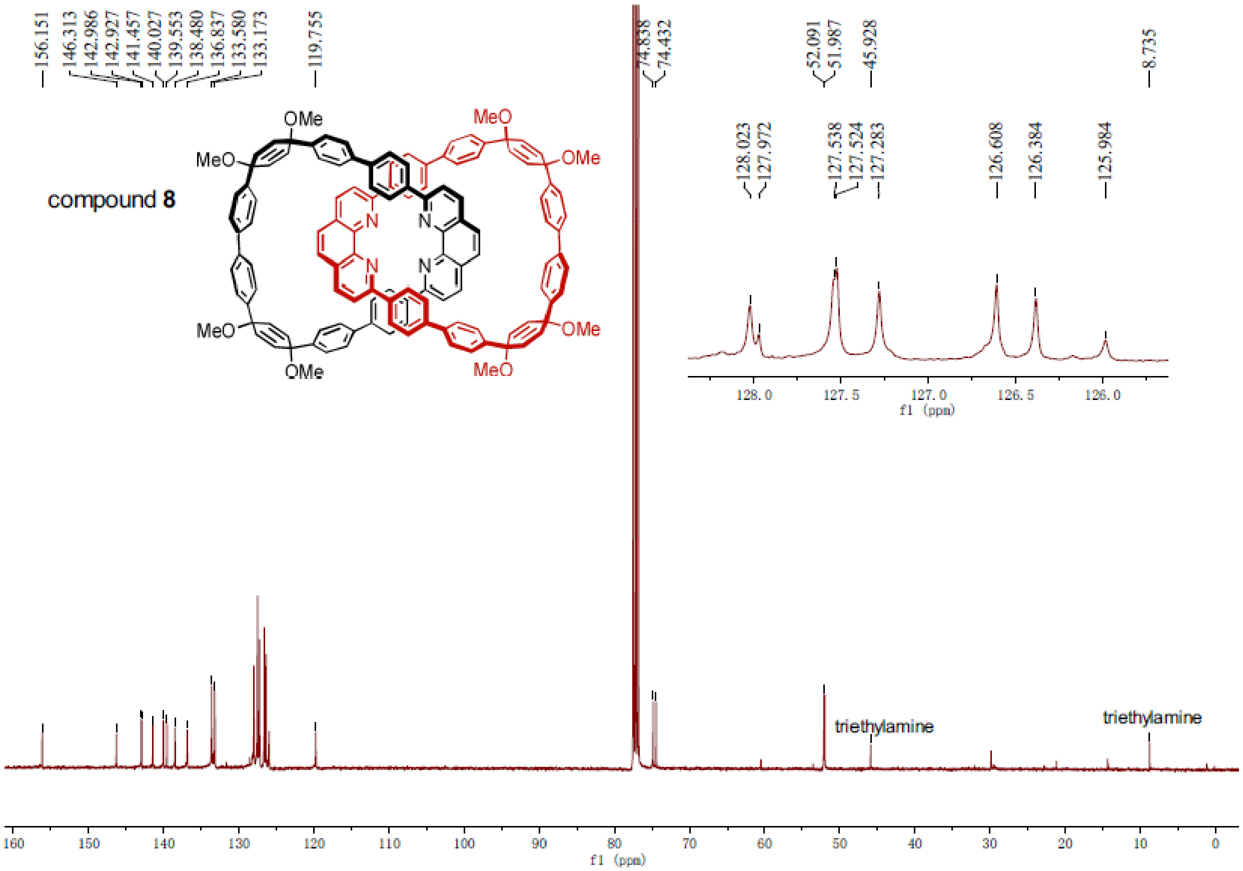

Supplementary Figure 30. ^{13}C NMR spectra for compound **8**.

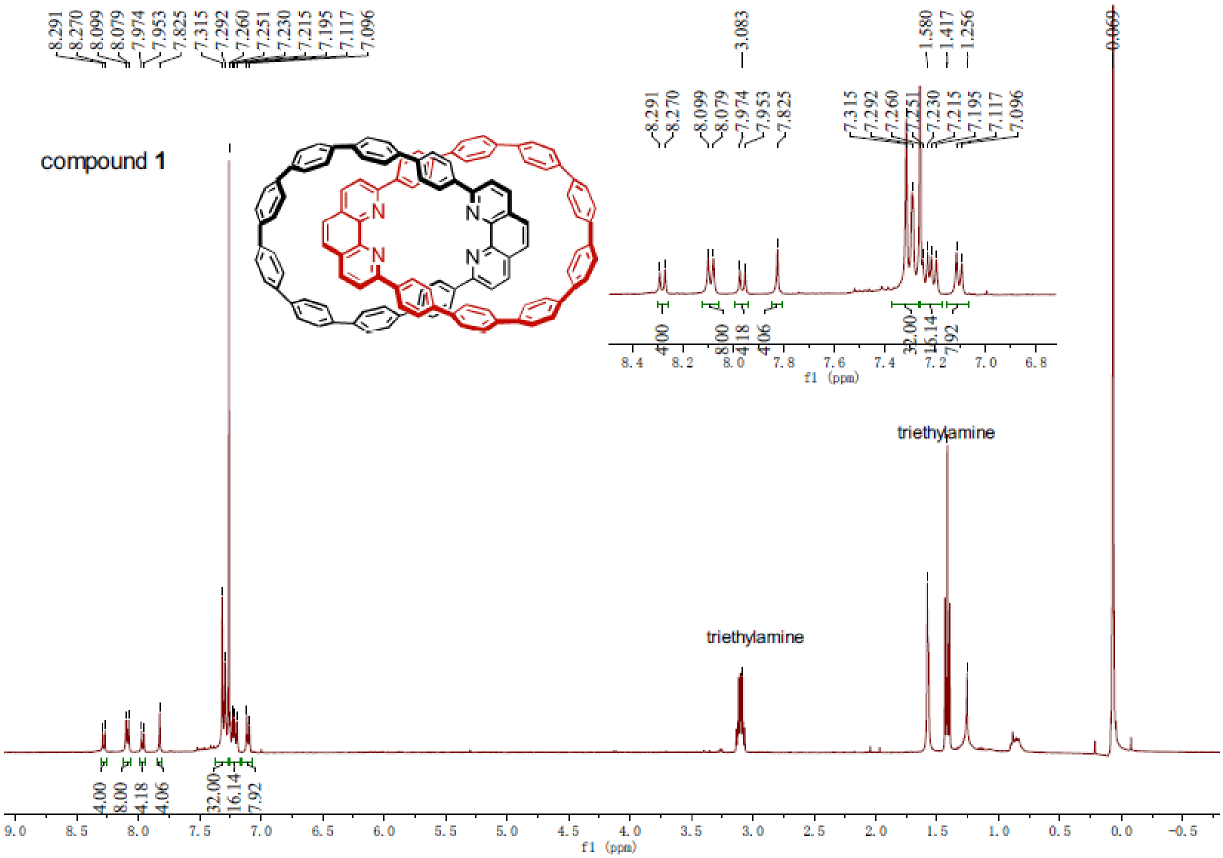

Supplementary Figure 31. ^{1}H NMR spectra for compound **1**.

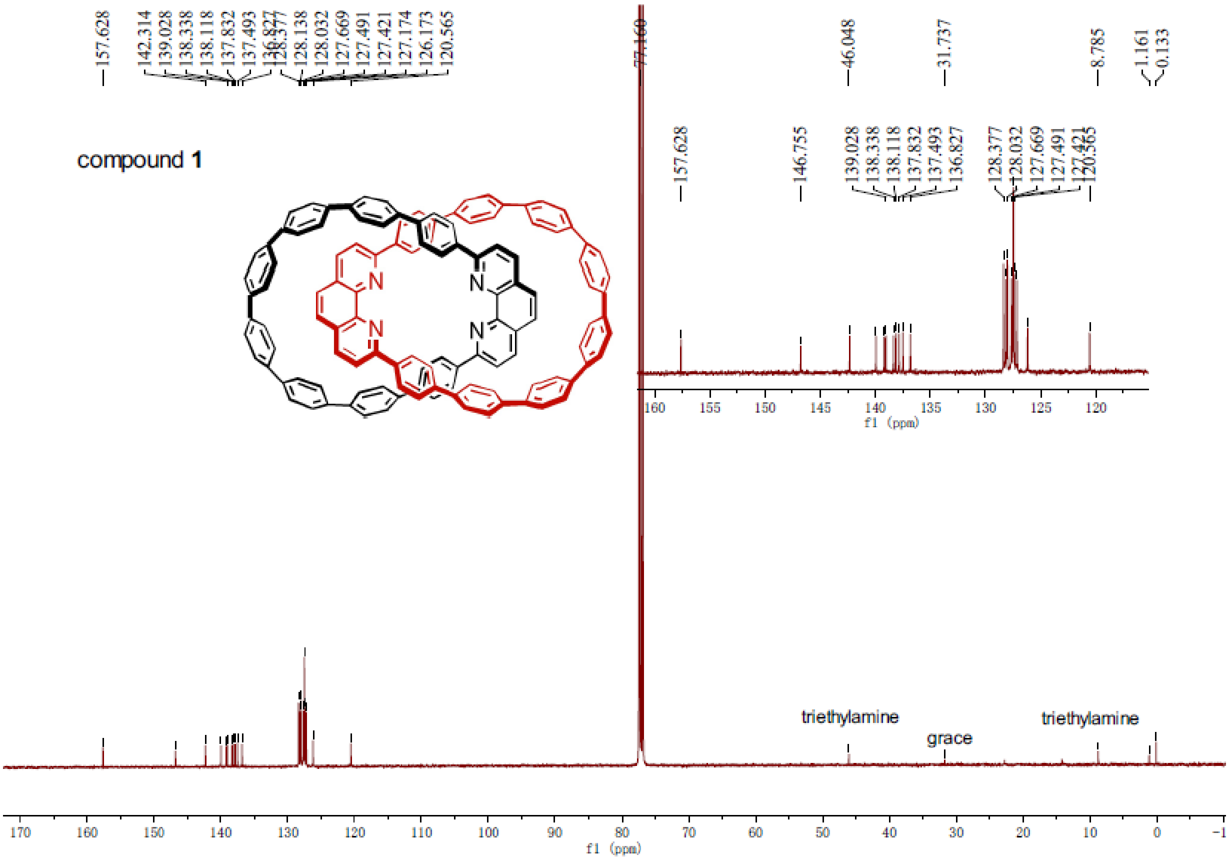

Supplementary Figure 32. ^{13}C NMR spectra for compound **1**.

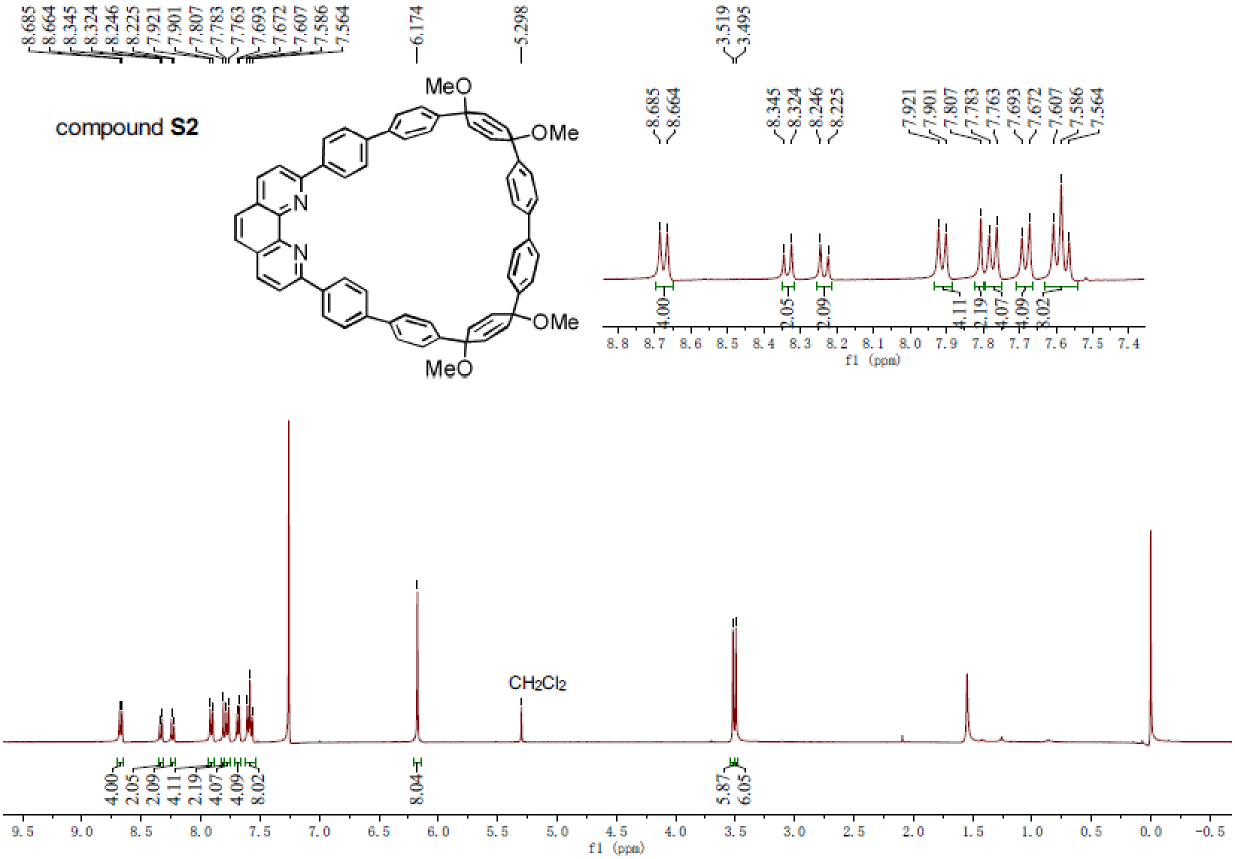

Supplementary Figure 33. ^{1}H NMR spectra for compound **S2**.

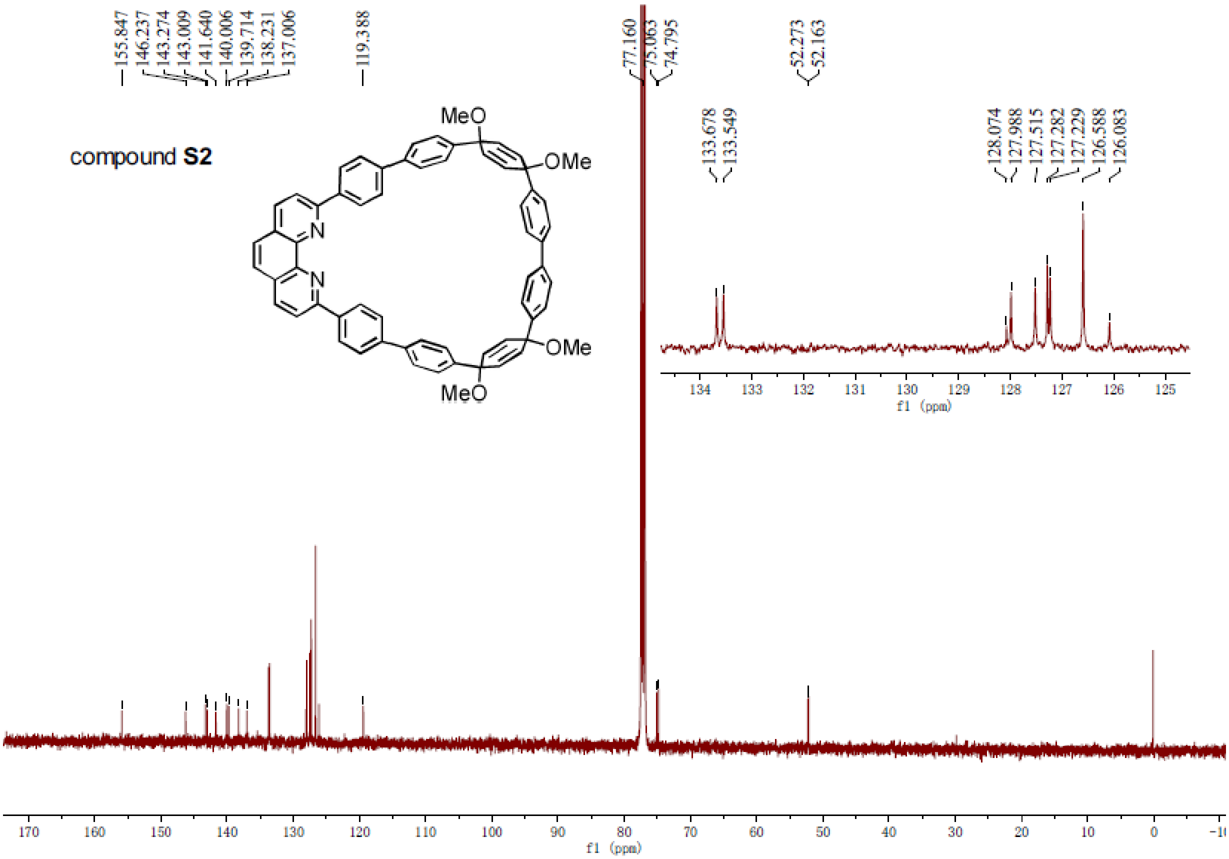

Supplementary Figure 34. ^{13}C NMR spectra for compound **S2**.

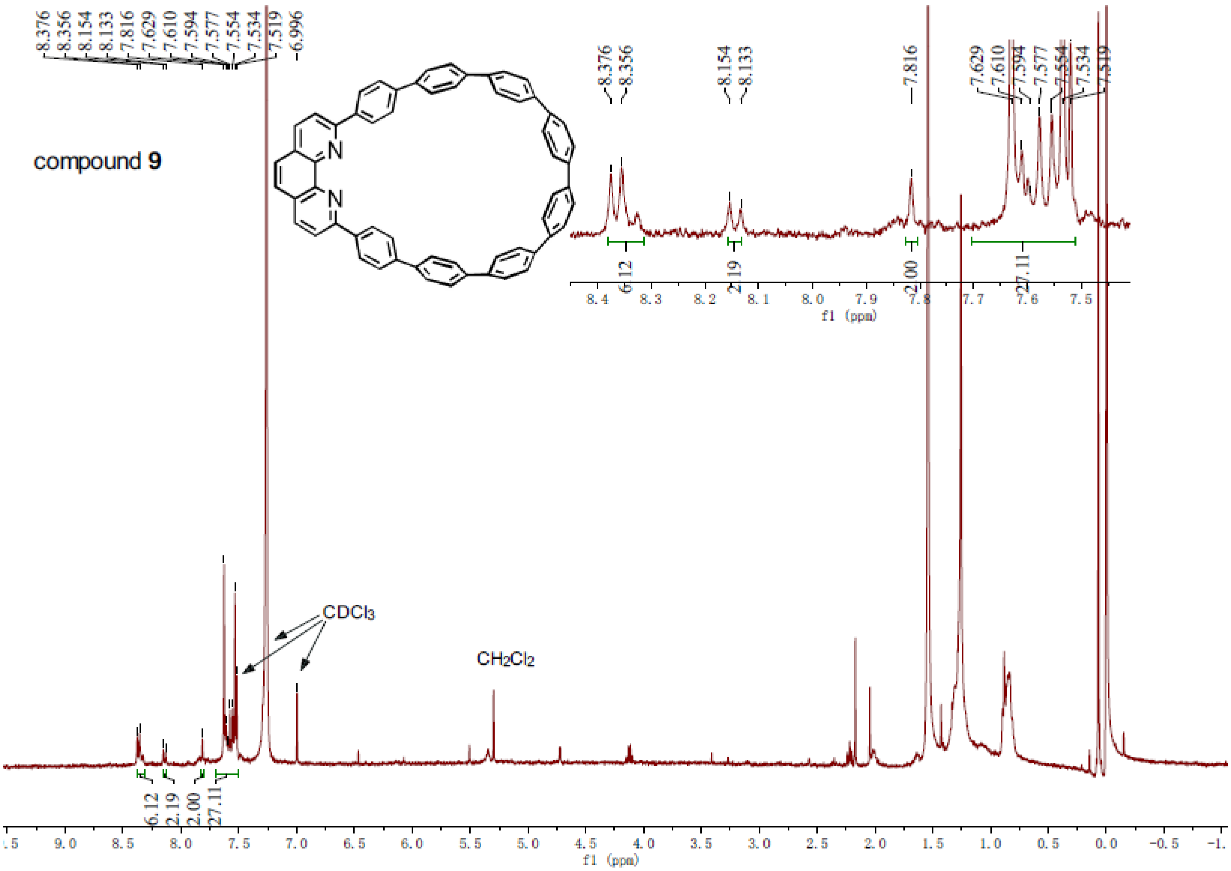

Supplementary Figure 35. ^{1}H NMR spectra for compound **9**.

Supplementary References

1. Frisch, M. J. et al. Gaussian 09, Revision E.01, Gaussian, Inc., Wallingford CT, 2016.
2. Zhao, Y., Truhlar, D. G. The M06 suite of density functionals for main group thermochemistry, thermochemical kinetics, noncovalent interactions, excited states, and transition elements: two new functionals and systematic testing of four M06-class functionals and 12 other functionals. *Theor. Chem. Acc.* **120**, 215-241 (2008).
3. Rassolov, V. A., Pople, J. A., Ratner, M. A., Windus, T. L. 6-31G* basis set for atoms K through Zn. *J. Chem. Phys.* **109**, 1223-1229 (1998).
4. Hehre, W. J., Ditchfield, R., Pople, J. A. Self—Consistent Molecular Orbital Methods. XII. Further Extensions of Gaussian—Type Basis Sets for Use in Molecular Orbital Studies of Organic Molecules. *J. Chem. Phys.* **56**, 2257-2261 (1972).
5. Becke, A. D. Density functional thermochemistry. III. The role of exact exchange. *J. Chem. Phys.* **98**, 5648-5652 (1993).
6. Krishnan, R., Binkley, J. S., Seeger, R., Pople, J. A. Self-consistent molecular orbital methods. XX. A basis set for correlated wave functions. *J. Chem. Phys.* **72**, 650-654 (1980).
7. Clark, T., Chandrasekhar, J., Spitznagel, Günther, W., Schleyer, P. v. R. Efficient diffuse function augmented basis sets for anion calculations. III. The 3-21+G basis set for first-row elements, Li–F. *J. Comput. Chem.* **4**, 294-301 (2004).
8. Frisch, M. J. et al. Gaussian 03, Revision E.01, Gaussian, Inc., Wallingford CT, 2004.
9. Keith, T. A., Bader, R. F. W. Topological analysis of magnetically induced molecular current distributions. *J. Chem. Phys.* **99**, 3669-3682 (1993).
10. Lu, T. & Chen, F. Multiwfn: a multifunctional wavefunction analyzer. *J. Comput. Chem.* **33**, 580-592 (2012).
11. Humphrey, W., Dalke, A., Schulten, K. VMD: Visual molecular dynamics. *J. Mol. Graphics* **14**, 33-38 (1996).
12. te Velde, G. *et al.* Chemistry with ADF. *J. Comput. Chem.* **22**, 931-967 (2001).
13. Fonseca Guerra, C., Snijders, J. G., te Velde, G. & Baerends, E. J. Towards an order-N DFT method. *Theor. Chem. Acc.* **99**, 391-403 (1998).
